# Neurochondrin drives colorectal cancer progression by modulating the PODXL–Ezrin axis and mitochondrial function

**DOI:** 10.1038/s41419-026-08747-5

**Published:** 2026-04-17

**Authors:** María Garranzo-Asensio, Elisa Carral-Ibarra, Ana Montero-Calle, Marta Gómez de Cedrón, Javier Velázquez-Gutiérrez, Susana Molina, Jana Dziakova, Rodrigo Sanz-López, Carmen Povès, Ana Ramírez de Molina, Javier Martínez-Useros, María Jesús Fernández-Aceñero, Rubén A. Bartolomé, Rodrigo Barderas

**Affiliations:** 1 Chronic Disease Programme (UFIEC), Instituto de Salud Carlos III, Madrid, Spain; 2 Escuela de Doctorado, Universidad Complutense de Madrid, Madrid, Spain; 3 Molecular Oncology Group, IMDEA Nutrition IMDEA Food, Madrid, Spain; 4 Instituto Investigación Clínico San Carlos (IdISSC), Hospital Clínico San Carlos, Madrid, Spain; 5 Translational Oncology Division, OncoHealth Institute, Health Research Institute Fundación Jimenez Diaz, Fundación Jimenez Díaz University Hospital, Universidad Autónoma de Madrid (IIS-FJD, UAM), Madrid, Spain; 6 Area of Physiology, Department of Basic Health Sciences, Faculty of Health Sciences, Rey Juan Carlos University, Madrid, Spain; 7 Department of Pathology, Hospital Clínico San Carlos, Madrid, Spain; 8 Departament of Legal Medicine, Psychiatry and Pathology, Universidad Complutense, Madrid, Spain; 9 Centro de Investigaciones Biológicas Margarita Salas, CSIC, Madrid, Spain; 10 CIBER Frailty and Healthy Aging, Madrid, Spain

**Keywords:** Metastasis, Colorectal cancer

## Abstract

Neurochondrin (NCDN) has been recently identified as overexpressed in liver metastatic colorectal cancer (CRC) cells compared to poorly metastatic isogenic counterparts. While its cellular function and role in cancer and CRC remain unknown, patient survival data indicate that elevated NCDN levels are associated with poor prognosis. After validating these observations in an independent patient cohort, we sought to investigate the role of NCDN in CRC progression using isogenic CRC cell models comprising poorly metastatic KM12C and liver-metastatic KM12SM cells, and poorly metastatic SW480 and lymph node-metastatic SW620 cells. Stable silencing of NCDN expression in these cell lines resulted in a significant reduction in their tumorigenic and metastatic properties in vitro. Furthermore, in vivo assays using nude mice and the KM12 cell model demonstrated that NCDN is essential for tumor initiation, growth, and liver metastasis of CRC cells. Proteomic profiling of NCDN-silenced cells uncovered a set of dysregulated proteins associated with cell adhesion, invasion, cell death, and differentiation, and mitochondrial dysfunction, emerging NCDN-PODXL-EZR axis as a critical mediator of CRC progression and involved in liver metastasis. Our findings position NCDN and their associated dysregulated proteins as drivers of CRC progression and promising targets for further investigation.

## Introduction

As of 2022, colorectal cancer (CRC) ranks as the third most common and the second deadliest cancer type worldwide [1]. CRC is a heterogeneous disease with slow progression influenced by both genetic and environmental factors. Numerous genetic abnormalities in well-characterized cancer genes have been associated with CRC [2]. However, the environmental contributors to CRC carcinogenesis are diverse and significantly amplify the disease’s heterogeneity [3]. Regardless of their origin, genetic and environmental risk factors promote CRC development by primarily targeting colon epithelial cells [4, 5]. This leads to genomic and epigenomic instability, which facilitates the accumulation of genetic and epigenetic alterations. These alterations disrupt the function of oncogenes and tumor suppressor genes, driving the malignant transformation of colon epithelial cells [3, 6, 7]. Subsequently, these transformed cells acquire invasive properties, enabling intravasation into the bloodstream, extravasation, and the colonization of distant organs, forming metastatic niches [8, 9].

Liver metastasis is the primary cause of CRC mortality, accounting for ~90% of CRC-related deaths. Unveiling the main drivers of CRC liver metastasis could facilitate the development of targeted therapeutic strategies. Quantitative proteomic analyses of cell lines sharing the same genetic background but exhibiting different metastatic features are potent tools for uncovering clinically relevant mechanisms [10–14]. The isogenic KM12 CRC cell system and the isogenic pair SW480/SW620 are valuable approaches for the study of CRC progression and metastasis [11, 15–18]. The KM12 model, derived from a stage II CRC patient classified as Duke’s B (T3, N0 in TNM classification), comprises three epithelial cell lines: KM12C, characterized by low metastatic potential; KM12SM, which exhibits enhanced liver metastatic ability; and KM12L4a, which shows liver and lung metastasis. This model is particularly valuable for studying the latest stages of metastasis, including liver colonization and survival [11, 19, 20]. Additionally, the SW480 and the SW620 cell pair have been demonstrated to be useful for the analysis of CRC lymph node colonization [16, 18].

Building on these insights, recent studies have leveraged in-depth proteomics to analyze both the secretome and the spatial proteome of the KM12 system [11, 19, 20], revealing over 1200 proteins differentially expressed in KM12SM cells compared to KM12C cells, and providing a comprehensive view of CRC liver metastasis biology [15, 16], in comparison to proteins differentially associated to lymph node involvement in CRC through the analysis of the SW480/SW620 cells [19]. Among them, key molecules such as VEGFA, ERBB2, EGFR, MMP7, AIP, CDH17, or IL13Rα2 have been implicated in CRC progression, and have shown a strong correlation with patient-derived data [11]. These analyses have highlighted the potential of quantitative functional proteomics to identify novel metastasis-associated proteins and pathways. Given that approximately 20% of all human reference proteins remain poorly characterized [21, 22], these efforts have significant potential to reveal new targets of intervention.

In this study, we focused on the analysis of neurochondrin (NCDN), a protein with limited functional characterization in cancer and, specifically, in CRC. Although NCDN has been primarily associated with neurodevelopment, proteomic data from the KM12 cell model revealed its marked upregulation in the highly metastatic KM12SM and SW620 cells [15], prompting us to investigate its potential role in CRC progression and liver metastasis. To this end, NCDN expression was silenced in highly metastatic KM12SM and SW620 cells, and in the poorly metastatic KM12C and SW480 CRC cells, and the effects on tumorigenic and metastatic potential were evaluated. In vitro and in vivo functional experiments, together with the in silico analysis of CRC datasets, confirmed that NCDN is involved in CRC progression and metastasis. Mechanistic proteomic analyses further revealed a strong link between NCDN, podocalyxin-like protein (PODXL), and EZR (ezrin), implicating this axis in cytoskeletal remodeling and tumor cell dissemination. These findings underscore the relevance of NCDN in CRC progression and identify PODXL and ezrin as key downstream effectors and potential therapeutic targets in advanced CRC.

## Materials and methods

### CRC samples and in silico analysis of prognostic value

Paired tumoral and non-tumoral optimal cutting temperature (OCT)-embedded frozen tissue samples were obtained from the Hospital Clínico San Carlos (IdISSC) biobank after the approval of this study on biomarker discovery and validation (CEI PI 13_2020-v2, and CEI PI 90_2023-v2) by the Ethical Review Boards of the Instituto de Salud Carlos III, and Hospital Clínico San Carlos (Madrid, Spain), which belong to the National Biobank Network (ISCIII) cofounded with FEDER funds. Written informed consent was obtained from all patients. Tissue samples were collected using a standardized sample collection protocol, histopathologically analyzed, and stored at −80 °C until use (Table S1).

The prognostic value of NCDN was assessed by Kaplan–Meier curves using the best cut-off method for separating high and low-expression populations, log-rank test for significance, and two different datasets (COAD dataset from the TCGA and GSE17538 dataset). GEPIA2 tool (http://gepia2.cancer-pku.cn/) was used for the in silico analysis of NCDN prognostic value with COAD TCGA dataset (318 tumor samples) [23], whereas for GSE17538 database (232 CRC samples with clinicopathological data) the data were normalized using Bioconductor’s Affymetrix package and transformed into z-scores.

### Tissue microarray assembly, staining, and analysis

Tissue samples from 131 CRC patients diagnosed at the Hospital Clínico San Carlos were used to assemble a tissue microarray (TMA) following approval by the Ethical Committee. An MTA-1 tissue arrayer (Beecher Instruments) was used to gather the biopsies from the previously sectioned samples (1 mm in diameter), which were then paraffin-embedded. Staining was performed in 3 µm sections. TMAs were scanned with the NanoZoomer scanner (Hamamatsu Photonics), and ×40 and ×80 images were processed with the NDP.view 2 software (version 2.7.25).

For the statistical analysis of the TMA, only samples that retained perfect integrity were analyzed. Each individual sample was assigned different parameters: staining localization, staining intensity (0–3), and percentage of stained cells (0–100). Sample quality allowed for the processing of 42 of the samples. To stratify samples, the values obtained for the staining intensity and percentage of stained cells were multiplied. ROC curves and statistical analysis of the data were obtained with GraphPad and R.

### CRC cell culture and stable NCDN silencing

KM12C, KM12SM, and KM12L4a cell lines were obtained from I. Fidler’s laboratory (MD Anderson Cancer Center). KM12 cells were expanded in the laboratory to generate a large batch of working aliquots, which were subsequently stored in liquid nitrogen. For each experiment, cells were thawed and maintained in culture for no more than 10 passages. These three cell lines were not authenticated in our laboratory. The isogenic SW480 and SW620 cells, as well as the HT-29, Caco-2, RKO, SW48, and Lim1215 CRC cell lines, were obtained from the American Type Culture Collection (ATCC, Manassas, VA, USA) cell repository.

Cells were grown and maintained in Dulbecco’s Modified Eagle Medium (DMEM, Lonza)—high glucose 25 mM, or low glucose 10 mM—supplemented with 10% fetal bovine sera (FBS, Sigma Aldrich), L-glutamine (Lonza), and penicillin/streptomycin (Lonza) (complete medium) at 37 °C and 5% CO_2_, and regularly monitored for mycoplasma contamination. For luciferase expression, cells were infected with lentiviral particles containing the luciferase gene reported (KM12C_vLuc and KM12SM_vLuc), as previously done [24]. NCDN depletion was achieved by lentiviral infection, individually using four different short hairpin RNAs (shRNAs) provided from Merck in the KM12 cell model or shRNA 4 in SW480/SW620 CRC cells. Selection of silenced or control cells was achieved at 1 µg/ml puromycin (Innovagen) while selected cells were maintained at 0.5 µg/ml puromycin following established protocols [25]. As shRNA control vector, a scrambled shRNA (shCT, #SHC002, Merck) was used.

### In vitro cell-based assays

For proliferation assays, cells were collected and seeded in p96 well plates. For each condition, 2000, 5000, and 10,000 cells were seeded in triplicate in 100 µl of complete medium. After 72 h, 50 µl of 3 mg/ml MTT (3-(4,5-dimethylthiazol-2-yl)-2,5-71 diphenyltetrazolium bromide, Sigma Aldrich) in complete growth medium was added into each well and left for 1 h at 37 °C. Cell culture media was then removed, and 50 µl of DMSO (Merck) was added onto the wells to dissolve formazan crystals. Absorbance was measured at 570 nm using the Spark multimode microplate reader (Tecan Trading AG). For adhesion assays, plates were coated overnight with 0.4 µg/mm^2^ of Matrigel (Sigma Aldrich) in 100 µl NaHCO_3_ while cells were left overnight in FBS-free medium. The next day, cells were harvested with 4 mM EDTA-PBS and labelled with 1 mg/ml BCECF (Sigma Aldrich) for 1 h at 37 °C. After labelling, cells were re-suspended in 50 µl of adhesion media (0.5% BSA in DMEM without phenol red) and seeded into the coated plates previously blocked for 2 h at 37 °C, also using adhesion media. In all cases, 100,000 cells were seeded in triplicate. After 2 h at 37 °C, unattached cells were washed away through various PBS washes and gentle tapping of the plates. Lastly, cells were lysed with 50 µl of 1% SDS, and their emitted fluorescence at 535 nm was measured after excitation at 436 nm.

Colony formation assays were performed as previously reported [26] using 25,000 cells for each condition. For cell-viability assays, annexin V-FITC binding was assessed in cells by labelling for annexin V-FITC and propidium iodide (PI) according to manufacturer’s instructions (MACS; Miltenyi Biotec). As control, untreated cells were assessed. Fluorescence intensity was measured using the BD FACSCanto (BD Biosciences) using standardized gating strategies.

### Microfluidics assay

For cell attachment assays, BJ-hTERT fibroblasts or GFP-expressing HUVEC (Human Umbilical Vein Endothelial Cells) cells were seeded to confluence into µ-Slide VI^0.4^ ibiTreat wells. Then, 150,000 shCT, and shNCDN KM12C and KM12SM cells treated with CellTracker Orange CMTMR (Invitrogen) (Table S2) were injected using a 20 µl/min continuous flowrate in 0.8 mm tubes and after extensive washing of the cell channels at a rate of 50 µl/min. Cell passage and attachment were monitored using the Thunder Imager (Leica). Images were taken at 10× and then analyzed with the LASX program. Attached cells were counted at final time.

### Cell bioenergetics

Oxidative phosphorylation and aerobic glycolysis were indirectly quantified by monitoring the oxygen consumption rate (OCR) and extracellular acidification rate (ECAR), respectively, using the extracellular flux bioanalyzer Seahorse, according to established protocols [19].

For the MitoStress assay, 20,000 cells were seeded in 10% FBS-DMEM the day prior to the experiment. Then, the media was changed to 10 mM glucose, 2 mM glutamine, and 2 mM pyruvate in non-buffered DMEM pH 7.4 (MitoStress glucose) or 10 mM galactose, 2 mM glutamine, and 2 mM pyruvate in non-buffered DMEM pH 7.4 (MitoStress galactose). Cells were then kept for 1 h at 37 °C in the absence of CO_2_. Basal OCR was then measured, and the modulators of the respiratory chain were added sequentially following the MitoStress kit: 2 μM oligomycin, 0.8 μM FCCP, and 0.5 μM rotenone/antimycin A. OCR was measured three times after the injection of each drug.

For the GlycoStress assay, 20,000 cells were also seeded in 10% FBS-DMEM the day prior to the experiment. Then, the media was changed to 2 mM glutamine in non-buffered DMEM pH 7.4, and cells were kept 1 h at 37 °C without CO_2_. Then, following the Glycolysis Stress kit, the different compounds were injected sequentially: 10 mM glucose, 0.5 μM oligomycin, and 50 mM 2-deoxy-D-glucose (2-DG). ECAR was measured three times after the injection of each compound.

### Immunofluorescence

For immunofluorescence (IF) staining, 50,000 cells were seeded into 12-well chambers in complete medium 10% FBS-DMEM, glutamine, and penicillin/streptomycin at 37 °C and 5% CO_2_. After cell attachment, the cultured media were removed, and cells were extensively washed before incubating them for 24 h in serum-free DMEM. For focal adhesion assessment, cells were maintained in serum-free medium before seeding them into previously Matrigel-coated wells (1:1000). Then, cells were fixed for 10 min at 37 °C with 4% paraformaldehyde (PFA) and washed three times with PBS prior to cell permeabilization using 0.1% Triton-**×**100 in PBS for 15 min at 37 °C and blocking of unspecific unions using 10% FBS-PBS (1 h at room temperature -RT-). Then, wells were incubated overnight at 4 °C with selected antibodies at optimized concentrations (Table S2). After antibody removal and washing, wells were incubated with the corresponding fluorophore-conjugated secondary antibodies and/or FITC-labelled Phalloidin (Table S2) for 1 h at RT. Lastly, wells were incubated for 10 min with Hoechst for nuclear staining prior to washing the wells and mounting the samples. IF images were taken using the SP5 Confocal microscope (Leica) and analyzed with the LASX software.

### In vivo experiments

The Ethical Committee of the Instituto de Salud Carlos III (Spain) approved the protocols used for the experimental work with mice after approval by the OEBA ethical committee (Proex 285/19). All experiments were conducted following standardized protocols and following good clinical practices for the use of laboratory animals. Animals were monitored daily for signs of distress and to determine that tumor sizes never exceeded the limits allowed by the approved protocols. Sample size was determined following ethical principles and considering expected effect sizes from previous studies. The 3 Rs principle was followed in all cases to minimize animal use. Animals were randomly assigned to experimental groups, and data collection was performed without blinding.

For liver homing experiments, 10^6^ KM12SM_vLuc shCT and KM12SM_vLuc shNCDN cells in 100 µl of PBS were injected into the spleen of female nude mice (*n* = 2 per group). After 24 h, the mice were sacrificed, and their spleen and liver were collected prior to RNA extraction. For tumor formation assays, 10^6^ cells (KM12SM_vLuc shCT, and KM12SM_vLuc shNCDN) in 100 µl of PBS were injected subcutaneously in the flank of nude mice (*n* = 6 for each group). Tumor growth was monitored and measured three times per week throughout the duration of the experiment upon tumor formation. Additionally, tumor growth rate was calculated for each condition. For liver metastasis assays, 1.5 × 10^6^ cells (KM12SM_vLuc shCT, and KM12SM_vLuc shNCDN) were injected into the spleen of nude mice (*n* = 5 for each group, although one mouse was lost during the second surgery in the shNCDN group). After 24 h, the spleen was removed, and mice were inspected daily. Two weeks after intrasplenic cell inoculation, liver metastases were observed in isoflurane-anesthetized mice by luminescent activity of metastatic cells and nodules using luciferin (12.5 mg/kg, intraperitoneal injection). Luminescence was recorded with the IVIS in vivo imaging system (Perkin Elmer) every 7 days up to 30 days after intrasplenic cell injection.

### Protein extraction and western blot analysis

After cellular pellet retrieval using 4 mM EDTA-PBS, proteins were extracted using 1 ml of RIPA buffer (Sigma-Aldrich) supplemented with protease and phosphatase inhibitor cocktails (1:100 dilution, MedChemExpress). Cells were disrupted through consecutive passes through 16 G and 18 G needle syringes. Then, solutions were clarified by 10 min centrifugation at 12,000 × *g* and 4 °C. Protein concentration was measured through the tryptophan method [20, 27]. To evaluate the quality of the protein extracts, 10 µg of each sample was loaded onto 10% PAGE-SDS gels under reducing conditions. After protein separation, protein quality was observed by Coomassie blue staining. For western blot (WB) analysis, after protein separation through 10% PAGE-SDS, proteins were transferred into nitrocellulose membranes for 90 min at 100 V. Membranes were then blocked with 3% BSA in 0.1% Tween-PBS (PBST) for 1 h at RT prior to their overnight incubation with indicated antibodies at optimized dilutions (Table S2) at 4 °C. Next day, membranes were extensively washed with PBST and incubated 1 h at RT with HRP-labelled secondary antibodies (Table S2). After washing the membranes with PBST, proteins were visualized with SuperSignal West Pico Maximum Sensitivity Substrate using the Amersham ImageQuant 800 (Cytiva) (see Supplementary Material for full and uncropped WB).

### RNA extraction and cDNA synthesis

For RNA extraction, the cellular pellet was disaggregated for 5 min with 1 ml of NZYol (NZYTech). For RNA extraction of the tissue samples (*N* = 14), the tissue was disaggregated using the TissueLyser II (Qiagen). Then, 200 µl of chloroform was added, and the samples were centrifuged for 10 min at 12,000 × *g* and RT for phase separation. The aqueous phase containing the RNA was further purified using the NZY Total RNA extraction kit (NZYTech) following manufacturer’s instructions. For cDNA synthesis, the NZY First-Strand cDNA Synthesis Kit, separate oligos (NZYTech), with 1 µg of RNA, was used following manufacturer’s instructions.

### Semi-quantitative (PCR) and quantitative PCR (qPCR) analysis

For gene expression analysis using PCR, 0.8 µl of cDNA was used in a mixture of 5× buffer HF, 1 M betaine, 1.6 µl of dNTPS, 1 µl of 10 µM forward primer, 1 µl of 10 µM reverse primer, and 0.1 µl of Phusion enzyme in a final volume of 20 µl. PCR products were loaded into 1.5% agarose gels for visualization. For qPCR analysis, a mixture of 5 µl of TB Green Premix Ex Taq II (Tli RNase H Plus) (Takara), 0.2 µl of 10 µM forward primer, 0.2 µl of 10 µM reverse primer, and 2 µl of 1:10 cDNA was used in a final volume of 10 µl. The qPCR was performed at 60°C and 45 cycles in a Light Cycler 480 (Roche). Primer sequences are indicated in Table S3.

### TMT labelling and LC-MS/MS analysis

For identification and quantification of the NCDN-associated proteome, the TMT 10-plex Mass Tag labeling kit (Thermo Fisher Scientific) protocol was followed as indicated by manufacturers using 10 μg of protein extracts per condition [20, 27]. Prior to protein extraction, cells were incubated for 48 h in FBS-free DMEM at 95% confluence. Briefly, a total of 10 µg of each individual or pooled protein extract in 100 µl of RIPA were reduced with 10 µl 100 mM TCEP for 45 min at 37 °C and 600 rpm and alkylated with 11 µL of 400 mM chloroacetamide for 30 min at RT, 600 rpm, and in darkness. After that, samples were incubated with 100 µl of SeraMag magnetic beads mix (50% hydrophilic beads - 50% hydrophobic beads, Cytiva) and 200 µl of 100% acetonitrile (ACN) for 35 min at RT and 600 rpm. Then, the supernatants were discarded, and the magnetic beads with the bound proteins were washed twice with 70% ethanol and once with ACN. Lastly, supernatants were discarded, and proteins were overnight digested at 37 °C and 600 rpm with 0.5 µg of porcine trypsin in 100 µL of 200 mM HEPES, pH 8.0. Next, samples were sonicated twice, and the supernatants containing digested peptides were collected. Samples were separately labeled in duplicate with the different Tandem Mass Tags in two incubation steps of 30 min at RT and 600 rpm with 10 µl of reagent per incubation, and a final incubation with 10 µl of 1 M glycine, pH 2.7, for 30 min at RT and 600 rpm. Finally, the content of the 10 tubes was pooled together and dried under vacuum prior to peptide separation using the High pH Reversed-Phase Peptide Fractionation Kit (Thermo Fisher Scientific). For separation, dried peptides were reconstituted in 300 µl of 0.1% Trifluoroacetic acid (TFA) in H_2_O_mq_ and columns were equilibrated twice with 300 µl of ACN and twice with 300 µl of 0.1% TFA in H_2_O_mq_. Then, peptides were loaded to the columns, washed twice with 300 µl of 0.1% TFA in H_2_O_mq_, and separated in 12 fractions of 300 µl each in 0.1% triethylamine, 2.5–100% ACN gradient. Then, fractions were dried under vacuum and stored at −80 °C until analysis by LC-MS/MS. Samples were reconstituted in 10 µl of 0.1% formic acid (FA) prior to LC-MS/MS analysis. TMT experiments were analyzed on an Orbitrap Exploris 480 mass spectrometer (Thermo Fisher Scientific) equipped with FAIMS Pro Duo interface technology following laboratory’s standard guidelines [20]. For FAIMS, a gas flow of 4.6 L/min and CVs -70V and -50V were used [27]. For data analysis MaxQuant (version 2.1.3) was used using standardized workflows. Mass spectra files were searched against Uniprot UP000005640_9606.fasta Homo sapiens (human) 2019 database (20,962 protein entries) using the MS2 type reporter ion. Precursor and reporter mass tolerance was set at 4.5 ppm and 0.003 Da, respectively. TMTs data normalization and sample loading (SL) normalization was performed with R Studio (version 4.1.1) according to established protocols (https://github.com/pwilmart). Data analysis was performed with R Studio using the packages “dplyr”, “tidyverse”, “edgeR”, “limma”, “rstatix”, and “ggplot2”, and moderated t-statistics were used for the statistical analysis of differential expression in TMT data. Contaminant proteins were removed, data were filtered to retain proteins identified in at least 70% of samples per group, and missing values were imputed by random draws from a Gaussian using the “imputeLCMD” R package. Proteins identified with 1 or more unique peptides, a fold change of ≥1.5 or ≤0.67 and *p* value < 0.05, were selected as upregulated and down-regulated, respectively.

### Immunoprecipitation coupled to LC-MS/MS

Immunoprecipitation of NCDN was performed following established protocols [20, 28]. First, 50 μL of beads (Protein-G Plus Agarose; SCBT) were equilibrated with 500 μL of RIPA buffer (centrifuging 1 min at 1000 × *g* at 4°C and removing the supernatant). Beads were re-suspended in 50 μL of RIPA buffer. As a precleaning, 40 μL of beads were incubated with 1.5 mg of KM12C and KM12SM protein extracts for 4 h at 4°C in the rotatory agitator. Then, the precleaned extract was incubated overnight with 0.5 μg of NCDN monoclonal antibody (Table S2) prior to binding the immunocomplexes to the remaining resin for 3 h at 4 °C in the rotatory agitator. The resin used for precleaning was used as a negative control. The beads were washed out through consecutive centrifugations (1000 rpm, 1 min, 4 times) using RIPA buffer. For protein isolation, 1.5% β-mercaptoethanol in Cracking Buffer (without bromophenol blue) was added and incubated at 95 °C for 5 min. Lastly, samples were centrifuged (2000 rpm, 2 min) to obtain the protein eluates. For LC-MS/MS analysis, eluates from in solution protein digestion were dried under speed-vacuum and dehydrated samples were reconstituted in 10 µL 0.1% formic acid and 3 μL of each sample were injected four times using the Vanquish Neo UHPLC System (Thermo Fisher Scientific). For liquid chromatography (LC), samples were loaded into a precolumn PepMap 100 C18 3 µm, 75 µm × 2 cm Nanoviper Trap 1200BA (Thermo Fisher Scientific) and eluted in an Easy-Spray PepMap RSLC C18 2 µm, 75 µm × 50 cm (Thermo Fisher Scientific) heated at 50 °C. The mobile phase flow rate was 300 nL/min using 0.1% FA H2O (buffer A) and 0.1% FA in 80% ACN (buffer B). The 1 h gradient was: 2% buffer B for 5 min, 2–42% buffer B for 60 min, 42–95% buffer B for 1 min, and 95% buffer B for 10 min. For the MS/MS analysis, 1900 V of liquid junction voltage and 280 °C capillary temperature were used for ionization. The full scan method employed a m/z 400–1500 mass selection, an Orbitrap resolution of 120,000 (at m/z 200), an automatic gain control (AGC) value of 300%, and a maximum injection time (IT) of 100 ms. For MS2, the 15 most intense precursor ions were selected for MS/MS fragmentation with a normalized collision energy of 30, and MS/MS scans were acquired with a 100 m/z first mass, an AGC target of 100%, a resolution of 15,000 (at m/z 200), an intensity threshold of 1 × 10^4^, an isolation window of 1.6 m/z, and the maximum IT was set to Auto. Charge state screening was enabled to reject unassigned, singly charged, and greater than or equal to seven protonated ions. A dynamic exclusion time of 20 s was used to discriminate against previously selected ions. MS data analysis was performed with MaxQuant (version 2.1.3) using standardized workflows. Mass spectra *.raw files were searched against Uniprot UP000005640_9606.fasta Homo sapiens (human) 2024 database (20,418 protein entries). After obtaining the list of identified proteins, the proteins were filtered according to the following criteria. First, contaminant proteins (i.e. keratins) were removed, and proteins with peptides present in the negative controls and proteins without peptides present in the positive samples were excluded. Then, proteins with less than two MS/MS were removed. For validation by WB, the IP protocol was repeated with the corresponding monoclonal and polyclonal NCDN antibodies (Table S2).

### Bioinformatic analysis

The expression pattern and correlation of selected genes were studied using the TCGA database and UALCAN (https://ualcan.path.uab.edu/) and GEPIA 2 web resources (http://gepia2.cancer-pku.cn/#index). For survival analysis, data from the Human Protein Atlas were used (https://www.proteinatlas.org/). The STRING database (https://string-db.org) was used to analyze interactions among the dysregulated proteins identified. Additionally, it was employed to explore potential NCDN interactors by examining the network of proteins identified in the immunoprecipitation assay and expanding it through the inclusion of two additional nodes together with the PODXL-ezrin axis. The MultiExperiment Viewer software (MeV) was used to visualize the expression levels of dysregulated proteins. Statistical analyses were performed using Microsoft Excel and GraphPad.

### Statistics

Sample size was chosen based on practical considerations and prior studies investigating similar outcomes [20, 24, 29, 30]. For the in vitro and cell bioenergetic assays, at least three technical and three biological replicates were performed. For the microfluidic assays, two technical replicates were performed, and at least 14 different images were taken for cell attachment analysis. For TMT analysis, two biological replicates were performed. Variance between groups was evaluated descriptively by comparing standard deviations across groups to ensure robustness. Unpaired *t* test was used to establish statistical results.

## Results

### NCDN expression in CRC and evaluation of its role as prognosis marker

**Fig. 1:**
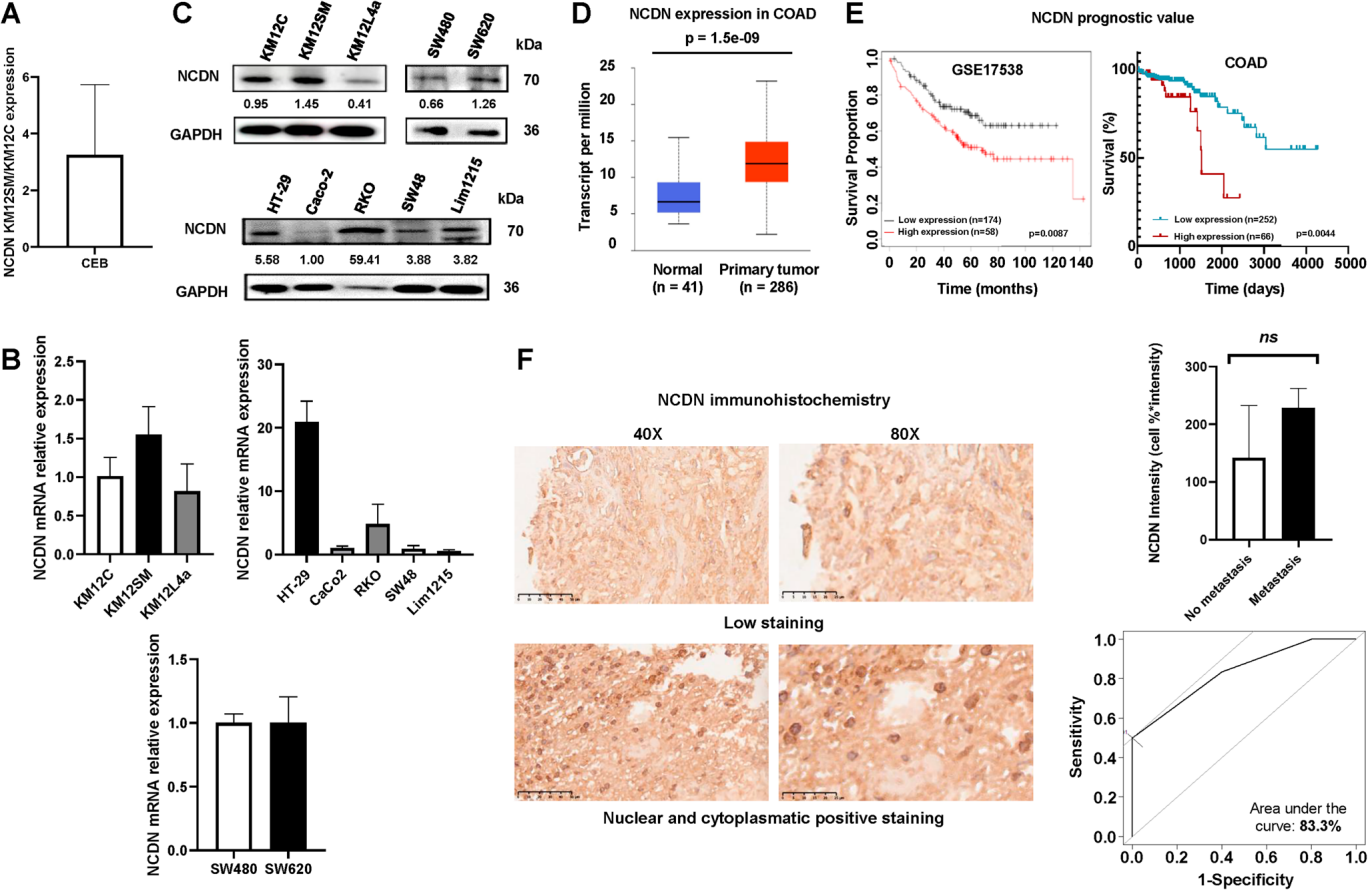
NCDN expression levels in CRC. **A** Previous proteomic study found that NCDN was overexpressed in highly metastatic to liver CRC cells (KM12SM) compared to the isogenic, non-metastatic KM12C cells in the CEB (citoplasmic) cellular compartment [15, 16]. Values are represented as the mean ± SD of the heavy/light (forward experiment) and light/heavy (reverse experiment) ratios. **B** Quantification of mRNA levels of NCDN in other CRC cells. **C** Protein levels of NCDN in various CRC cell models by WB confirmed the results obtained by qPCR and proteomic studies, and showed that the SW620 (metastatic to lymph nodes) cells had higher NCDN protein levels than their isogenic, low-metastatic SW480 cells. **D** TCGA data showed higher levels of NCDN in primary tumors compared to the normal tissue of COAD patients. **E** Higher expression of NCDN is associated with a lower survival rate in both the publicly available GSE17538 dataset and TCGA COAD dataset. **F** Higher NCDN staining intensity in the nucleus and cytoplasm was found in metastatic compared to non-metastatic CRC patients. Data are represented as mean ± SD. NCDN staining could discriminate between metastatic and non-metastatic tumors with an AUC of 83.3%. COAD colon adenocarcinoma, TCGA The Cancer Genome Atlas program; AUC area under the curve; *p* values correspond to *t* test: **<0.01, ns not significant.

Previous spatial proteomic studies regarding protein abundance and localization of liver metastatic KM12SM CRC cells compared to KM12C poorly-metastatic CRC cells [15] identified neurochondrin (NCDN) as a protein overexpressed in the cytoplasm (CEB) of KM12SM cells (Fig. 1A). Here, we have focused on characterizing the role of NCDN in CRC and on elucidating its functional significance in CRC progression.

First, a panel of CRC cell lines was analyzed by qPCR and WB to verify the proteomics findings on the KM12 cell model (Fig. 1B, C), and to assess whether NCDN expression was restricted to these cells. Remarkably, ∼50% upregulation of NCDN at mRNA and protein levels by qPCR and WB, respectively, was confirmed in highly liver metastatic KM12SM cells in comparison with the isogenic poorly metastatic KM12C cells. In contrast, liver and lung metastatic KM12L4a cells showed lower NCDN expression at mRNA and protein level. Furthermore, NCDN expression levels were also ~50% higher in the isogenic lymph-node metastatic SW620 cells compared to the non-metastatic SW480 cells at the protein level, while mRNA levels remain similar. Additionally, elevated NCDN protein levels were also detected in the poorly differentiated RKO cell line. At the protein level, the low-aggressive and low-metastatic HT-29, Caco-2, and Lim1215 cell lines showed similar expression patterns, with HT-29 seemingly having elevated NCDN levels, also corroborated by qPCR (Fig. 1B, C) [31].

Next, the dysregulation of NCDN expression at mRNA level was confirmed in CRC samples. We evaluated its expression levels in the TCGA using the colon adenocarcinoma (COAD) dataset and its association with patients’ survival. We found that NCDN was overexpressed in the primary tumor tissue compared to the paired normal tissue *(p* = 1.5e-9) (Fig. 1D). Statistically significant overexpression was also observed when analyzing the data in the COAD database according to the stage of the disease (Fig. S1). Moreover, although the overall overexpression of NCDN in the rectum adenocarcinoma dataset was not significant (Fig. S1), statistical significance was observed when comparing data from normal and stage IV samples (*p* = 2.4e-2) (Fig. S1). Furthermore, Kaplan–Meier survival analyses in two independent public cohorts comprising a total of 550 CRC patients were conducted. High NCDN expression was significantly associated with reduced overall survival in the GSE17538 cohort (*p* = 0.0087). Consistently, in the COAD TCGA dataset, patients with advanced CRC (stages III–IV) and higher NCDN expression showed a significantly poorer survival probability (*p* = 0.0044), further supporting the association of NCDN with adverse clinical outcomes (Fig. 1E). Additionally, to further evaluate its clinical relevance at protein level, a TMA composed of 42 samples from metastatic and non-metastatic CRC patients was analyzed. A higher nuclear and cytoplasmic NCDN staining trend (*p* = 0.07) was observed in metastatic patients compared to non-metastatic patients positive for nuclear and cytoplasm NCDN staining (Fig. 1F), whose ROC curve analysis showed an area under the curve (AUC) of 83.3% comparing the metastatic and non-metastatic CRC patients.

Collectively, these findings confirm the overexpression of NCDN in CRC and its association with advanced stages, extending previous proteomic observations from cell lines to colorectal tumor tissues. This broader validation supports the association of NCDN with CRC progression, suggesting a functional role in the disease beyond the KM12 cell model.

### NCDN depletion decreases the tumorigenic and metastatic properties of CRC cells

**Fig. 2:**
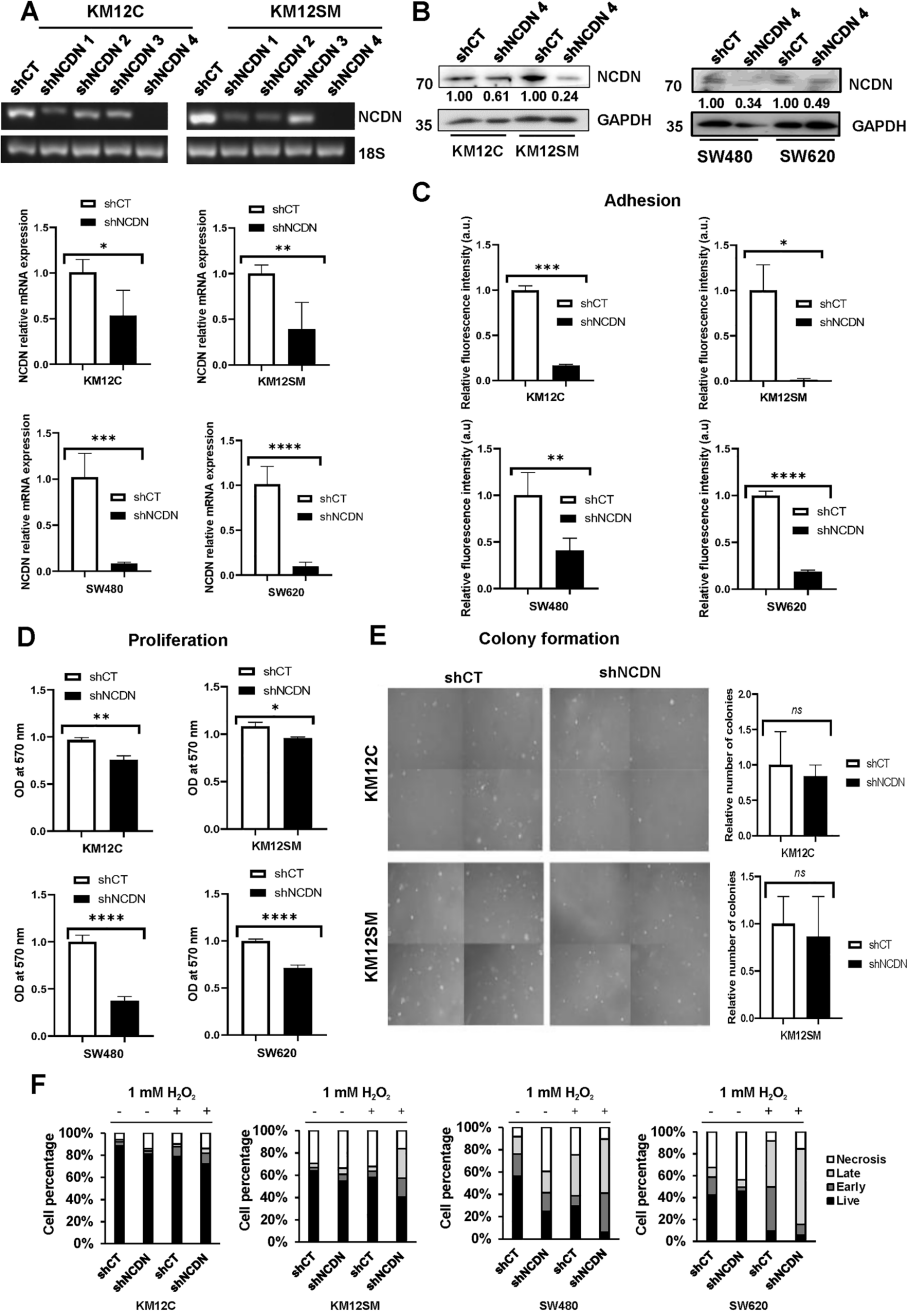
Functional effects in the tumorigenic and metastatic properties of KM12C (poorly metastatic) and KM12SM (metastatic to liver) CRC cells upon NCDN depletion. **A** NCDN depletion by shRNA was verified by semi-quantitative and quantitative PCR analyses. **B** NCDN protein levels upon depletion with shNCDN 4 were assessed by WB. **C** KM12C, KM12SM, SW480, and SW620 cells significantly lost most of their adhesion capacity to matrigel upon NCDN depletion. **D** KM12C, KM12SM, SW480, and SW620 cells significantly reduced their proliferation capacity with the depletion of NCDN levels. **E** Both the relative number of colonies and their size were reduced but not significantly in KM12C and KM12SM CRC cells upon NCDN depletion. **F** Cell status assessment showed a decrease in cell viability to oxidative stress upon stable NCDN depletion in KM12C, KM12SM, SW480, and SW620 CRC cells in the presence or absence of 1 mM H_2_O_2_. *p* values correspond to *t* test. *<0.05: **<0.01; ***<0.001; ****<0.0001, ns not significant.

To address this question, NCDN was silenced in KM12C and KM12SM cells stably expressing luciferase by preparing stable shRNA transfectants. As control, cells infected with a control shRNA were also prepared (shCT). NCDN depletion was confirmed by PCR, qPCR, and WB (Fig. 2A, B). Both analyses showed that less than 25% of the original protein expression levels were detected after knockdown with shRNA#4 (shNCDN 4) on KM12SM cells and about 50% on KM12 C cells.

Then, we analyzed the effect of NCDN depletion on the tumorigenic and metastatic properties using the stable shNCDN 4 NCDN-depleted CRC cells, as they showed the highest reduction in NCDN expression, in comparison with shCT in both the metastatic and non-metastatic cells (Fig. 2C–E). NCDN depletion significantly decreased the adhesion and proliferation capabilities of KM12C and KM12SM cells, whereas a non-significant decrease in colony-forming capacity to form colonies was observed. Results were especially noticeable in the adhesion of liver metastatic KM12SM CRC cells. Additionally, to test whether NCDN might somehow modulate cell survival, KM12 cells were subjected to apoptosis assays. NCDN depletion produced an increase in the percentage of necrotic cells in KM12C and KM12SM cells. In response to oxidative stress induced by 1 mM H_2_O_2_, a higher percentage of NCDN-depleted KM12C cells were either apoptotic or necrotic compared to control cells (Fig. 2F). Moreover, in KM12SM cells, the cell viability also showed this trend, with a marked decrease in live cells, and an increase in late apoptotic cells but not necrotic cells (Fig. 2F).

Additionally, to verify that the changes in the tumorigenic and metastatic properties of the cells associated with NCDN depletion were not specific to the KM12 cell model, we also evaluated the effect of the stable NCDN depletion in the isogenic SW480/SW620 cell pair using shRNA 4 in comparison with shCT (Fig. 2A, B). Upon confirmation of NCDN depletion by shRNA 4 in the SW480/SW620 cells by qPCR and WB, cell-based assays were performed. As observed in the KM12 model, the depletion of NCDN decreased the adhesion and proliferation capacity of the cells (Fig. 2C, D). Moreover, SW480 cells reduced their viability upon NCDN depletion (Fig. 2F), whereas in SW620 cells, a higher percentage of necrotic cells was observed upon NCDN depletion. Additionally, under oxidative stress conditions after the addition of 1 mM H_2_O_2_, the effects were more pronounced in the SW480 cells, with a marked reduction in cell viability in NCDN-depleted cells. In contrast, in SW620 cells, an increase in late apoptosis and necrosis upon NCDN depletion was observed, but with a similar percentage of live cells (Fig. 2F).

Collectively, NCDN depletion somehow disrupted the tumorigenic properties and survival signaling cascades in CRC cells, which might impair in vivo the ability of the liver metastatic KM12SM cells to grow, to establish metastatic niches, and to proliferate at the metastatic sites.

### NCDN depletion in KM12SM cells provokes a decrease in tumor growth, and liver homing and colonization in mice

**Fig. 3:**
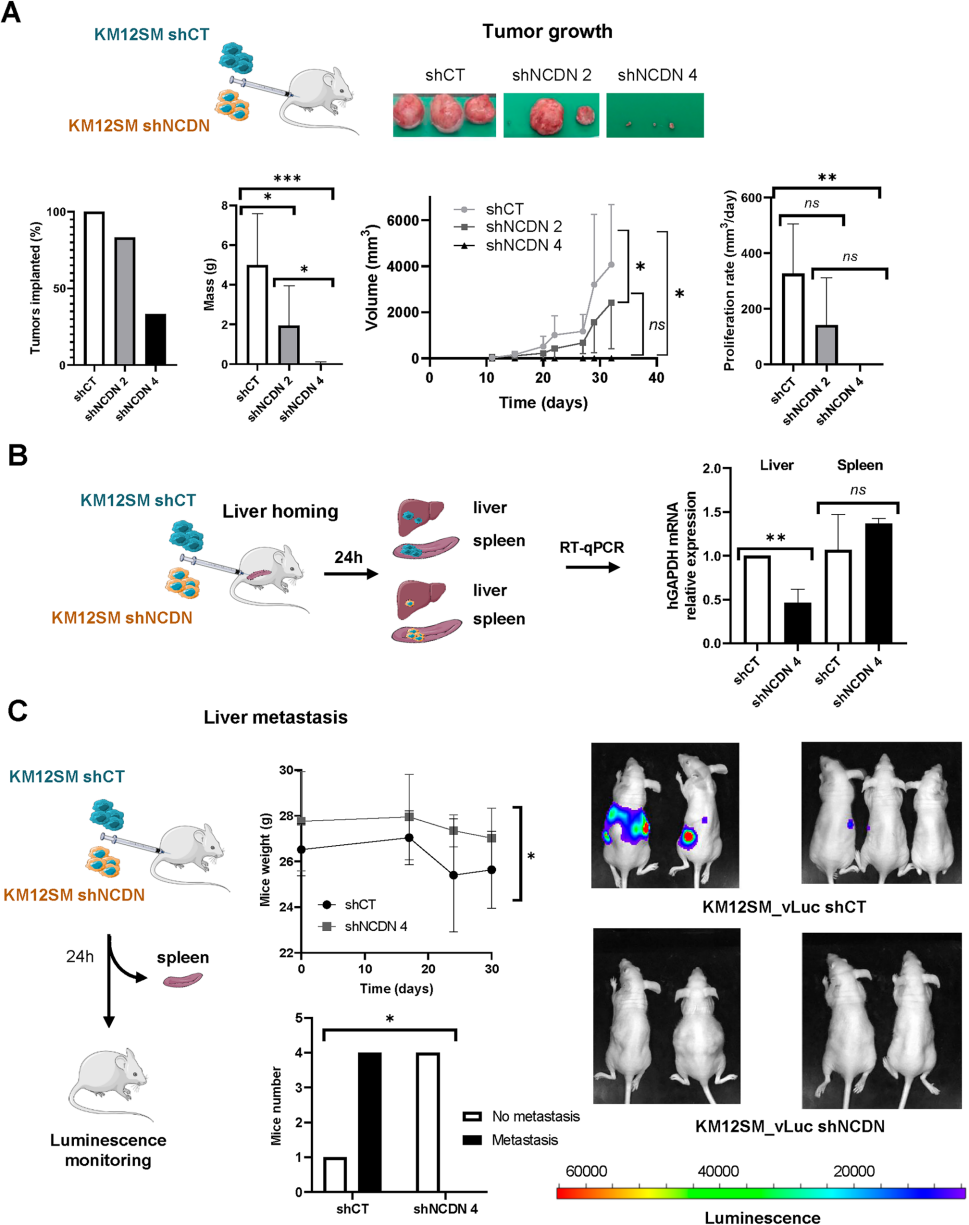
Liver homing and metastatic effects in vivo upon NCDN depletion in CRC cells. **A** Tumor formation and tumor size were dependent on NCDN expression. After injecting KM12SM-control, KM12SM-shNCDN 2 (intermediate NCDN expression), and KM12SM-shNCDN 4 (low NCDN expression) stably CRC cells, tumor implantation, mass, and volume were measured and determined to be proportional to NCDN expression. Tumor rate was also assessed. **B** The depletion of NCDN expression abrogated the migration of the KM12SM cells to the liver. **C** KM12SM-control and KM12SM-shNCDN four CRC cells expressing luciferase were injected into the spleen of the mice. After spleen removal, mice were weighted and metastasis occurrence was observed only in mice injected with control cells. *p* values correspond to *t* test. *<0.05: **<0.01; ***<0.001; ns: not significant. For tumor growth rate, differences were statistically significant for all cases except shCT vs shNCDN 2.

To investigate this issue and assess the role of NCDN in vivo in CRC progression and liver metastasis, we performed subcutaneous and intrasplenic injections of NCDN stably depleted KM12SM cells and their control counterparts (Fig. 3). Following the 3Rs principle to minimize animal use, in vivo experiments were conducted solely with KM12SM cells, as their liver metastatic capacity is well-established [19].

For tumor growth analysis, 1 × 10⁶ shCT (control) and shNCDN 4 KM12SM cells were subcutaneously injected in nude mice (Fig. 3A). To assess the effect of varying NCDN levels, we also included shRNA 2 KM12SM cells, which exhibit intermediate NCDN protein expression between shCT and shRNA 4 cells (Fig. 2A). Tumor growth strongly correlated with NCDN protein levels: while 100% of mice injected with shCT cells developed tumors, only 33.33% of mice injected with shNCDN 4 cells did, and tumors were significantly smaller. Mice injected with shNCDN 2 cells exhibited an intermediate tumor growth rate (tumor masses: 5.00 ± 2.58 g for shCT, 1.95 ± 2.01 g for shNCDN 2, and 0.033 ± 0.033 g for shNCDN 4) (Fig. 3A). The proliferation rate also correlated with NCDN levels (285.2 ± 188.8 mm³/day for shCT, 117.9 ± 163.1 mm³/day for shNCDN 2, and 0.20 ± 0.31 mm³/day for shNCDN 4).

Next, metastatic properties were assessed via liver homing (seeding) and liver metastasis (organ colonization) experiments. To reduce the number of animals, we focused on shNCDN 4 and shCT KM12SM cells. For liver homing, shCT and shNCDN KM12SM cells were injected into the spleen, and after 24 h, the spleen and liver were analyzed by qPCR using human GAPDH (hGAPDH) mRNA expression as a surrogate marker of the presence of KM12SM human cells in murine liver and spleen (Fig. 3B). Mice injected with shCT cells showed statistically significant higher levels of hGAPDH in the liver than mice injected with shNCDN 4 cells, confirming a reduced liver metastatic potential upon NCDN depletion.

Then, to evaluate liver metastatic colonization, shCT and shNCDN 4 KM12SM cells were injected into the spleen, which was removed 24 h after injection. Liver metastatic colonization was monitored via bioluminescence imaging after intraperitoneal luciferin injection (Fig. 3C). Significant results between groups were observed. Mice injected with shCT cells exhibited signs of distress, including significant weight loss, and four out of five mice developed liver metastases (ROI signal: 1898920 ± 2556929 total counts) at day 30 post-inoculation of cells. In contrast, no metastases or weight loss were observed in shNCDN 4-injected mice (ROI signal: 0 ± 0 total counts).

Collectively, our findings demonstrate a previously unreported association between NCDN and CRC liver metastasis, highlighting its critical role in tumor growth, metastatic seeding, and organ colonization.

### Proteomic analysis reveals dysregulated proteins associated with NCDN depletion

**Fig. 4:**
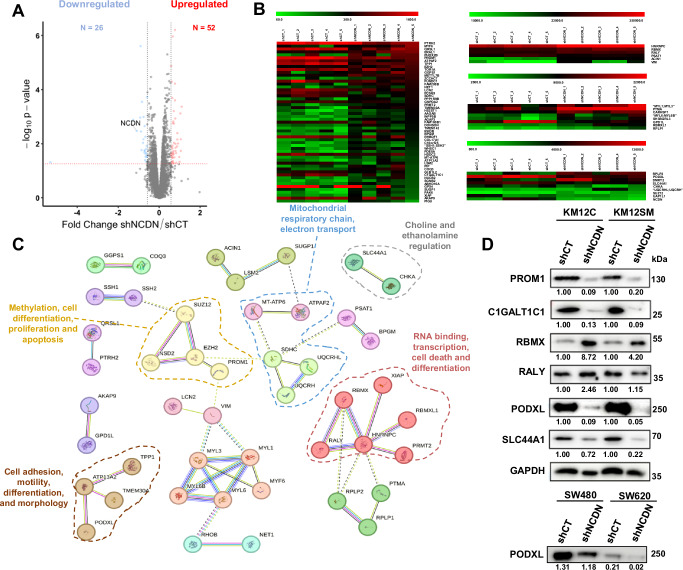
Analysis of the proteome revealed metastatic effectors associated with NCDN. **A** Comparison of the proteome associated with NCDN depletion revealed 26 downregulated and 52 upregulated proteins. **B** Clustering analysis of the expression pattern of the differentially expressed proteins showed a clear separation between control (shCT) and NCDN-depleted (shNCDN) conditions. **C** String analysis showed 13 interaction clusters associated with NCDN among the identified proteins. Highlighted clusters correspond to those selected for validation. **D** WB analysis validated the dysregulation of indicated proteins observed by proteomics upon NCDN depletion. For SW480 and SW620 CRC cells, PODXL abundance was normalized according to its corresponding GAPDH loading control depicted in Fig. 2B.

To elucidate the relationship between NCDN and CRC metastasis uncovering the biological pathways and processes modulated by NCDN, we conducted a TMT-based proteomic analysis to identify proteins dysregulated upon NCDN depletion. Protein extracts from stable shCT and shNCDN 4 KM12C and KM12SM cells in duplicate were quantified, trypsin-digested, and TMT-labeled prior to pH reverse-phase fractionation, and analyzed using an Orbitrap Exploris 480 mass spectrometer. Peptide and protein identification and quantification were performed using MaxQuant, and data analyzed using R (Fig. S2A). Principal component analysis was performed after proteomics data normalization (Fig. S2B). Interestingly, shCT cells (independent of their metastatic capacity), and shNCDN-depleted KM12C and KM12SM cells grouped separately, showing correct sample grouping (Fig. S2C). Statistical analysis establishing a fold change ≥1.5 or ≤0.67 and a *p* value ≤ 0.05 as indicative of dysregulated protein expression due to NCDN depletion revealed 26 and 52 proteins downregulated and upregulated, respectively (Fig. 4A, B, Table 1). Next, to gain further insight into the molecular context of NCDN, we performed a STRING analysis, which revealed six significant clusters of at least three interacting proteins each, indicating that NCDN depletion impact lysosomal activity, cell adhesion, methylation, apoptosis, ribosomal and RNA-binding proteins, cell morphology, mitochondrial function, and RNA processing (Fig. 4C). As initial validation of the proteomic results, selected dysregulated proteins from each pathway were confirmed by WB, showing consistent expression patterns with the proteomics data (Fig. 4D). Additionally, the dysregulation of PODXL was also observed in the SW cell model upon NCDN depletion (Fig. 4D).

**Table 1:** List of significantly dysregulated proteins upon NCDN depletion.

Protein	Fold change* (shNCDN/shCT)	Protein	Fold change* (shNCDN/shCT)
Upregulated proteins		Downregulated proteins	
HNRNPC	1.71	PODXL	0.53
RALY	1.58	SLC44A1	0.67
ACIN1	1.51	PROM1	0.56
CARHSP1	1.53	GPD1L	0.59
RBMX	1.71	TPP1	0.53
RBMXL1	1.56	C1GALT1C1	0.42
PTMA	1.71	PTRH2	0.61
RPLP2	2.15	NCDN	0.47
RPLP1	1.64	PCSK9	0.54
AGAP3	2.05	CHKA	0.63
METTL7B	1.73	ATPAF2	0.59
MYL1;MYL3	1.61	SDHC	0.58
VIM	1.59	GNPDA2	0.66
SUZ12	1.65	QRSL1	0.64
FAM208B	1.86	AKAP9	0.55
ZCCHC7	2.08	PSAT1	0.61
PDE8A	1.85	GLB1L2	0.61
DONSON	1.66	RHBDF1	0.61
PAK6	1.90	FAM160B1	0.64
WHSC1	1.74	LCN2	0.64
SH3BGRL3	1.57	TOR1A	0.65
DMRT2	1.83	HDDC2	0.63
ABHD16A	1.54	UAP1L1	0.66
HS2ST1	1.74	LSM2	0.62
INPP5B	1.56	FUNDC1	0.64
MYF6	2.08	GPX4	0.03
NUCB2	1.74		
BPGM	1.61		
TMEM143	1.60		
AAGAB	1.52		
TMEM30A	1.77		
SGMS2	1.57		
C18orf25	1.57		
MYL6;MYL6B	1.53		
ERAL1	1.62		
SSH1;SSH2	1.61		
MT-ATP6	1.50		
PRMT2	1.53		
COL17A1	1.55		
PIP	1.97		
RHOB	1.66		
ATP13A2	1.58		
PPP1R9B	1.57		
UQCRHL;UQCRH	1.58		
EZH2	1.57		
PHOX2B	2.00		
NET1	1.56		
PIGU	1.54		
COQ3	1.58		
GGPS1	1.79		
XIAP	1.65		
SUGP1	1.53		

*p value ≤ 0.05

**Fig. 5:**
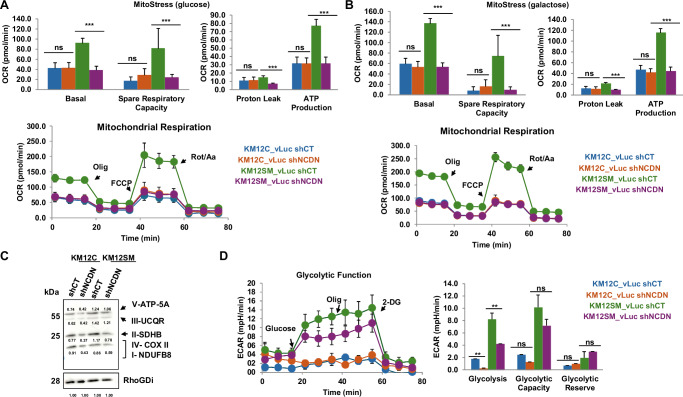
Analysis of the metabolic state associated with NCDN. **A** Assessment of NCDN expression on the oxygen consumption rate in the presence of glucose in control and NCDN-depleted cells. KM12SM_shCT cells showed a notably higher OCR level for basal conditions (91.6 ± 10.2 pmol/min), spare respiratory capacity (81.2 ± 40.6 pmol/min), and ATP production (76.9 ± 8.2 pmol/min) compared to KM12SM_shNCDN cells (38.2 ± 8.3 pmol/min; 24.1 ± 6.5 pmol/min, and 31.3 ± 8.5 pmol/min; respectively), KM12C_shCT (42.1 ± 11.0 pmol/min; 17.0 ± 8.4 pmol/min, and 31.4 ± 8.2 pmol/min; respectively), and KM12C_shNCDN depleted cells cells (42.5 ± 11.3 pmol/min; 28.4 ± 13.8 pmol/min, and 31.3 ± 7.5 pmol/min; respectively). **B** Assessment of NCDN expression on the oxygen consumption rate in the presence of galactose in control and NCDN-depleted cells. KM12SM_shCT cells showed a notably higher OCR level for basal conditions (136.2 ± 8.0 pmol/min), spare respiratory capacity (74.0 ± 11.4 pmol/min), and ATP production (115.1 ± 6.6 pmol/min) compared to KM12SM_shNCDN depleted cells (53.3 ± 8.3 pmol/min; 9.4 ± 3.9 pmol/min, and 43.8 ± 7.2 pmol/min; respectively), KM12C_shCT cells (59.0 ± 7.9 pmol/min; 7.6 ± 5.9 pmol/min, and 47.0 ± 7.7 pmol/min; respectively), and KM12C_shNCDN cells (53.1 ± 10.3 pmol/min; 16.0 ± 11.5 pmol/min, and 41.8 ± 8.5 pmol/min; respectively). **C** WB analysis of the different mitochondrial spare respiratory chain subunits of KM12C and KM12SM (control and NCDN-depleted) cells. RhoGDI was used as loading control. **D** Assessment of the glycolitic capacity of the cells measured as ECAR (mpH/min). KM12C-shCT cells showed higher levels of ECAR during glycolisis (1.8 ± 0.4 mpH/min) and when measuring glycolytic capacity (2.5 ± 0.6 mpH/min) than KM12C-shNCDN cells (0.3 ± 0.3 mpH/min and 1.3 ± 0.6 mpH/min). OCR oxygen consumption rate, ECAR extracellular acidification rate, Olig. oligomycin, FCCP Carbonyl cyanide-4 (trifluoromethoxy) Phenylhydrazone, Rot/An rotenone/antimycin, 2-DG 2-deoxy-D-glucose. *p* values correspond to *t* test. **<0.01; ***<0.001; ns not significant.

Among the clusters observed, the dysregulation of mitochondrial-associated proteins suggested mitochondrial dysfunction upon NCDN depletion. To explore this, we conducted MitoStress assays under glucose and galactose conditions. In KM12C cells, NCDN knockdown (shNCDN) did not significantly affect basal respiration, spare respiratory capacity, proton leak, or ATP production compared to controls (shCT), as measured by OCR. However, in KM12SM cells, shNCDN led to marked reductions in all these mitochondrial parameters (Fig. 5A, B), likely due to stronger NCDN depletion in KM12SM cells. WB analysis of various subunits of the mitochondrial respiratory chain further confirmed their downregulation in NCDN-depleted cells (Fig. 5C). Given that these findings pointed to a disruption of mitochondrial function on one of the major energy-yielding pathways, we also evaluated glycolysis using GlycoRate assays. Both KM12C and KM12SM NCDN depleted cells showed reduced glycolysis and glycolytic capacity relative to controls (Fig. 5D), in line with the observed upregulation of RBMX, a known inhibitor of aerobic glycolysis [32], in NCDN depleted cells. Overall, these results indicate that NCDN depletion disrupts key energy-yielding pathways, including oxidative phosphorylation and glycolysis, as predicted by the proteomic analyses, potentially impairing the tumorigenic capacity of CRC cells.

### NCDN modulates the PODXL-ERM axis in CRC cells

**Fig. 6:**
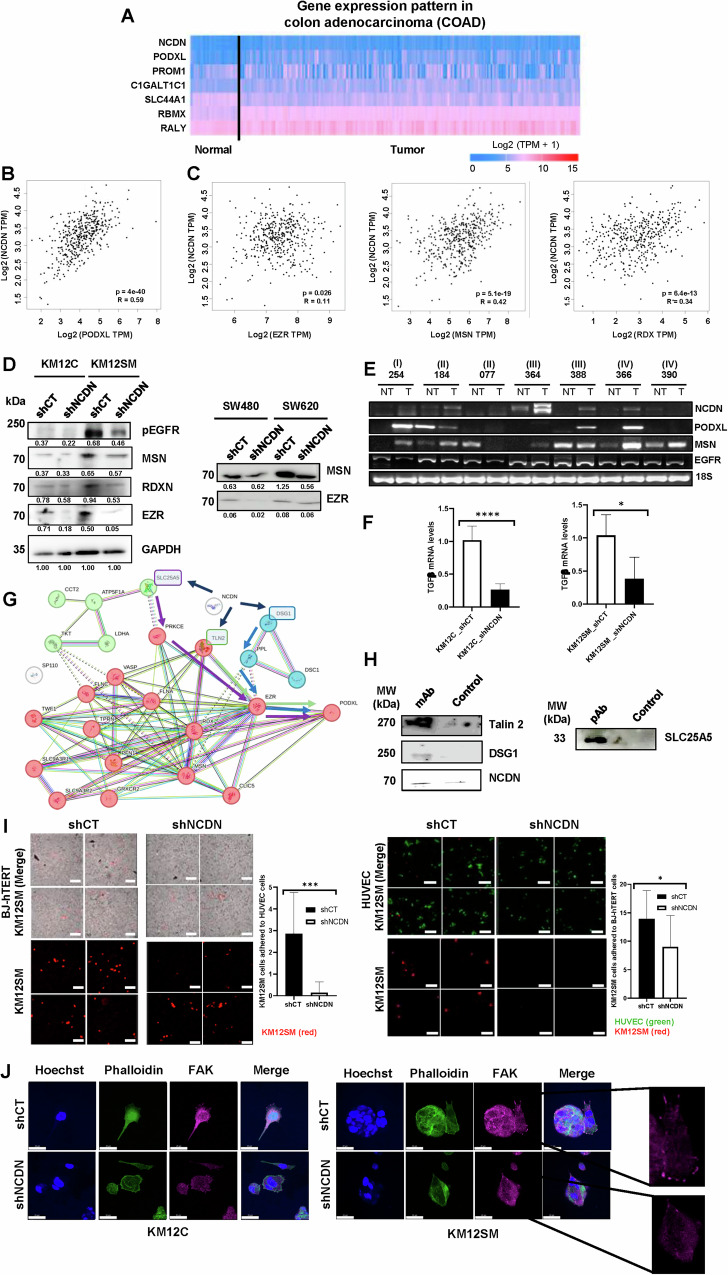
NCDN dysregulation impairs adhesion and alters key molecular pathways in liver metastatic CRC cells. **A** The expression patterns of selected dysregulated genes (PODXL, PROM1, C1GALT1C1, SLC44A1, RBMX, and RALY) with NCDN were analyzed in colon adenocarcinoma (COAD) samples. **B** PODXL showed a strong correlation pattern with NCDN in COAD samples (*R* = 0.59; *p* = 4e^−40^). **C** Correlation analysis of NCDN with the EZR-MSN-RDX triad in COAD samples. **D** WB analysis of the expression of the ERM protein family and pEGFR revealed reduced protein levels and diminished EGFR activation in cellular extracts of KM12C and KM12SM control and NCDN-depleted cells. MSN and EZR downregulation was also verified in SW480 and SW620 CRC cells. For the WB protein abundance, values of SW480 and SW620 CRC cells were normalized according to their corresponding GAPDH loading control depicted in Fig. 2B. **E** PCR analysis assessed mRNA levels of the selected genes and their correlation with NCDN transcript levels in human samples from patients at different stages of CRC. **F** Quantification of TGFβ-1 levels in KM12C and KM12SM (shCT and shNCDN) cells by qPCR. **G** String analysis showed the interaction network of the identified NCDN interactome, linking NCDN to the PODXL-ezrin axis. **H** Validation of the interaction between NCDN and Talin-2, DSG1, and SLC25A5 by WB analysis of IP performed with monoclonal or polyclonal antibodies against NCDN using the KM12 cell model. **I** Evaluation of the adhesive capacity of KM12SM cells (shCT and shNCDN) to endothelial (HUVEC) and fibroblast (BJ-hTERT) cells demonstrated reduced adhesion in NCDN-depleted cells. Size bars: 100 µm. **J** FAK and phalloidin immunofluorescence staining showed that KM12C cells rarely formed focal adhesions, whereas KM12SM cells readily formed them. Focal adhesions were significantly reduced in KM12SM_shNCDN cells. Size bars indicate 10 µm. COAD: colon adenocarcinoma. *p* values correspond to *t* test. *<0.05; ***<0.001; ****<0.0001; ns: not significant.

We next analyzed the expression of selected dysregulated proteins—chosen based on their known involvement in cancer progression and their marked downregulation upon NCDN depletion—and compared their expression patterns with that of NCDN in the COAD dataset from TCGA. This allowed us to assess their relevance in paired normal and tumor colorectal tissues and gain insight into the role of NCDN in CRC (Fig. 6A). Remarkably, PODXL showed a similar expression pattern with NCDN and a correlation of 0.59 (*p* = 4 × 10^−40^) (Fig. 6B). The cell-surface protein PODXL has been identified as a predictor of poor prognosis and distant metastasis in various cancer types, although its underlying mechanism of action remains unclear [33]. Recent studies suggest that PODXL contributes to tumor metastasis by enhancing extravasation efficiency in breast carcinoma cells [33], and by interacting via its intracellular domain with the ERM protein ezrin (EZR), a known mediator of metastasis [33–35]. Given this, we investigated whether NCDN expression correlates with ERM family members—EZR, moesin (MSN), and radixin (RDX)—which link the actin cytoskeleton to the plasma membrane and have all been implicated in cancer, including CRC progression [36–39]. Notably, NCDN showed significant correlations with all three ERM proteins (*p* < 0.026), with particularly strong associations observed for MSN and RDX (Fig. 6C).

Based on these results and previous studies [33], NCDN may influence liver metastasis of CRC cells by modulating PODXL through interactions with ERM (EZR, MSN, and RDX) proteins. To investigate this, we assessed the expression levels of ERM proteins in the KM12 cell model following NCDN depletion by WB (Fig. 6D). We also assessed the status of EGFR, since its expression has been significantly associated with that of PODXL in CRC [40]. NCDN depletion resulted in reduced ERM protein levels—especially EZR—and active EGFR (as determined by reduced pEGFR levels). Moreover, to verify that these results were not restricted to the KM12 cell model, the stably NCDN-depleted SW480/SW620 cell pair was analyzed. Importantly, the same downregulation for the ERM proteins was observed, supporting the observation that the decreased protein levels were associated with NCDN depletion and not cell-dependent (Fig. 6D). Additionally, to further corroborate whether these dysregulations occur in CRC patients, we evaluated the mRNA levels of NCDN and PODXL, MSN, and EGFR in paired normal and tumoral tissues from CRC patients (Fig. 6E). mRNA levels of MSN strongly correlated with NCDN expression in five out of seven patients. Moreover, EGFR mRNA levels remained unaltered, suggesting that NCDN depletion may affect EGFR activation rather than expression (Fig. 6E). Because PODXL has been implicated in metastasis via activation of TGFβ-1 [33], we also assessed TGFβ-1 mRNA levels. NCDN depletion led to a decrease in TGFβ-1 expression (Fig. 6F), reinforcing the impact of NCDN on this metastatic axis (Fig. 6F). However, it is worth noting that IF staining against PODXL and EZR in shCT and shNCDN cells did not show any differences in co-localization between PODXL and EZR dependent on NCDN expression (measured as Pearson’s coefficient), suggesting that NCDN’s relationship with PODXL’s metastatic properties is dependent on interacting mediators responsible for the interaction with ezrin (Fig. S3). To gain mechanistic insight, we performed immunoprecipitation assays followed by mass spectrometry (IP-MS) or WB to identify and validate NCDN-interacting partners potentially involved in the PODXL–ERM axis (Table S4, Fig. 6G). Among the identified proteins by IP-MS, we validated by WB the desmosome protein (DSG1), talin-2, and SLC25A5 as actual NCDN interactors involved in the PODXL-ERM axis (Fig. 6H). These findings suggest that NCDN modulates the PODXL-ezrin axis through a network of structural and adhesion-related interacting proteins able to modulate extravasation and focal adhesions.

### NCDN depletion impairs cancer cell extravasation and focal adhesion

To address this final issue and explore whether NCDN impacts extravasation and focal adhesion formation through the PODXL-ezrin axis [33], we conducted cell adhesion assays simulating extravasation and secondary organ adhesion through the different human compartments using endothelial (HUVEC) and fibroblast (BJ-hTERT) cells. As KM12SM cells are the ones able to metastasize to liver, these studies were focused on these cells. Importantly, we observed significantly reduced adhesion of NCDN-depleted KM12SM cells to both cell layers compared to shCT KM12SM controls (Fig. 6I). This effect was more pronounced in interactions with endothelial cells, supporting a role for NCDN in facilitating extravasation, as previously described for PODXL in breast cancer models [33].

Since extravasation requires effective focal adhesion formation, which mediates the interaction between cancer cells, the extracellular matrix, and surrounding cells [41], we investigated whether NCDN depletion impairs this process. KM12C cells inherently form few focal adhesions [42], and thus, the analysis was focused on KM12SM cells. Interestingly, IF staining of focal adhesion kinase (FAK) revealed a striking reduction in focal adhesion formation in NCDN-depleted cells, whereas shCT KM12SM cells showed robust FAK accumulation at adhesion sites (Fig. 6J).

Collectively, these findings position NCDN as a key modulator of the PODXL-ezrin axis in CRC metastasis. Through specific interactions with desmosomal proteins, talin-2, and SLC25A5, NCDN regulates downstream signaling events that promote extravasation, stable adhesion, and liver colonization. Its depletion impairs these processes, resulting in diminished tumor growth, adhesion, and metastatic outgrowth. Our results support a model in which the NCDN-PODXL-EZR network facilitates metastatic niche establishment and may represent a therapeutic vulnerability in advanced CRC.

## Discussion

Proteomics has emerged in the last decade as a crucial tool for studying metastasis, enabling the identification of key proteins involved in processes such as cell adhesion, invasion, and organ colonization [19, 20, 43–46]. Although the KM12 cell model does not fully replicate the complexity of spontaneous metastasis in human colon cancer, it offers a robust system for analyzing the underlying molecular mechanisms and critical proteins driving CRC progression and liver metastasis [11]. By providing valuable insights into the molecules involved in these processes, the proteomics analysis of this cell system, followed by functional validation studies, facilitates the identification of potential targets of intervention and enhances our understanding of CRC progression. In this work, we have focused on the functional analysis of the role of the barely known protein neurochondrin (NCDN) in CRC progression using the KM12 cell model, and the SW480/SW620 isogenic cell pair of CRC for confirmation of mechanistic insights. NCDN is physiologically expressed in the brain and is essential for normal brain development and homeostasis [47]. NCDN regulates neuronal morphology, synaptic function, and plasticity [47, 48]. Its dysfunction has been linked to neurological disorders, including schizophrenia and epilepsy [49]. In our study, we identified NCDN as a novel driver of CRC progression and associated with liver metastasis, offering new insights into the molecular mechanisms behind CRC progression. Besides, our study links the gut–brain axis with CRC, which represents a complex interplay between the nervous system and colorectal physiology [50], where neuronal signaling, gut innervation, and brain-derived factors influence intestinal homeostasis, inflammation, and cancer progression, demonstrating that neuronal-related proteins like NCDN play an unexplored role in CRC progression and tumor adaptability. Here, we have described an important role for NCDN in CRC. This conclusion is supported by the following observations: i) NCDN expression was higher in metastatic KM12SM and SW620 CRC cells compared to isogenic poorly-metastatic KM12C and SW480 cells, ii) NCDN depletion significantly reduced key tumorigenic properties such as adhesion, proliferation, and clonogenicity, iii) in vivo experiments demonstrated that NCDN depletion markedly reduced tumor growth, liver metastasis, and overall tumor burden, iv) proteomic analyses of the NCDN interactome using KM12 cells revealed that NCDN depletion downregulated PODXL and ezrin, both of which are associated with cell adhesion and metastasis, and that the PODXL-ERM axis is modulated through intermediate factors (i.e. desmosome proteins, SLC25A5, or talin-2), v) major findings observed in KM12 cells were replicated on SW480/SW620 CRC cells, and thus supporting the effect of NCDN depletion on CRC cells independently on the cells, vi) NCDN depletion reduced mitochondrial activity and altered metabolic pathways critical for CRC progression, and vii) analysis of patient samples using different datasets showed a correlation between NCDN expression and poorer clinical outcomes. These findings collectively demonstrate that NCDN is a key regulator of CRC progression and is associated with liver metastasis, highlighting its potential as a therapeutic target.

Regarding the interactome, PODXL was among the most downregulated proteins following NCDN depletion, suggesting that PODXL may act as a key mediator of CRC metastasis through the NCDN–PODXL–EZR axis, with ezrin being a well-established effector in metastatic progression [33]. These findings have not been previously reported. Interestingly, we confirmed by proteomics, PCR, and WB in cells and in CRC patients’ TMA that NCDN protein expression correlated with the protein expression of both PODXL and ezrin. However, no PODXL-ezrin colocalization by IF was observed. Notably, the downregulation of PODXL and ezrin correlated with decreased CRC cell adhesion to fibroblasts and endothelial cells. This aligns with prior findings in breast cancer, where reduced PODXL expression impaired cell extravasation and adhesion to metastatic niches [33]. Moreover, TGFβ-1 levels were also found to decrease in NCDN- and PODXL-depleted cells. As PODXL is known to promote epithelial-mesenchymal transition (EMT) via TGFβ signaling [51], these findings further support the critical role of NCDN in CRC metastasis. Furthermore, while this study provides valuable insights into mechanisms driving CRC progression, the precise relationship between NCDN and the PODXL-ezrin axis remains to be fully elucidated. Although NCDN depletion led to reduced levels of both PODXL and ezrin, no direct interaction between these proteins and NCDN was detected. However, IP-MS followed by mass spectrometry or WB identified desmosomal proteins, talin-2, and SLC25A5 as key intermediary factors potentially linking NCDN to the PODXL–ezrin axis. These proteins likely contribute to the observed phenotypes both in vitro and in vivo, and may play a broader role in CRC progression and metastasis alongside other yet-to-be-identified intermediates. Notably, the downregulation of LCN2 and LSM2 upon NCDN depletion also suggests their potential involvement in the regulatory network controlled by NCDN. LCN2 has been negatively implicated in the activation of the EMT even in CRC [52–56]. On the other hand, LSM2 is associated with poor prognosis and promotes cell proliferation, migration, and invasion in skin cutaneous melanoma [57]. Alongside PODXL, these proteins may represent additional mediators of CRC malignancy.

Moreover, the analysis of the proteome associated with NCDN depletion revealed other important cellular processes somehow related to NCDN. Downregulated proteins included GPX4 (glutathione peroxidase 4) and GNPDA2 (glucosamine-6-phosphate deaminase 2), which are implicated in redox balance and lipid/glucose metabolism [58, 59]. GPX4 is implicated in reducing fatty acid peroxidation, controlling inflammation, and apoptosis; and its upregulation has been associated with tumorigenesis, which may be related to the increased apoptosis found in our in vitro studies [58]. Meanwhile, GNPDA2 is related to the hexosamine signaling pathway, also playing a key role in lipid and glucose homeostasis [59]. Furthermore, proteomic analysis revealed the downregulation of SDHC, ATPAF2, and GPD1L -proteins critical for the production of ATP, ion homeostasis, and ROS regulation- upon NCDN depletion [60–62], which may explain the observed reduction in mitochondrial function and reduction in the proliferative capacity of the cells. All these results suggest that NCDN contributes to augmenting the buffering capacity of mitochondrial ROS, which is crucial to protect against apoptosis, programmed cell death, and anoikis, facilitating cancer dissemination [63]. However, its implication in these processes needs to be further evaluated since mitochondrial dysfunction may go beyond the evaluated processes. Additionally, proteins involved in the tumor microenvironment and cholesterol metabolism—PCSK9, RHBDF1, CHKA, and SLC44A1—were also downregulated in NCDN-depleted cells. These proteins are associated with proliferation, metastasis, hypoxic microenvironment regulation, and fibrotic stroma formation in CRC [64–68]. Thus, their reduced protein levels in NCDN-depleted cells suggest that NCDN might play an important role specifically for CRC malignancy. Of note, PROM1—a cancer stem cell marker that alters common abnormal processes in CRC [69]—and TPP1—implicated in telomerase processivity [70, 71]—protein expression levels were also lower in NCDN-depleted cells. Finally, among upregulated proteins, we identified RBMX, PIP, and ACIN1, all of which are associated with tumor suppression or apoptosis. RBMX suppresses tumor progression in bladder cancer [32], while PIP increased expression reduces cell proliferation and migration, and promotes apoptosis [72], and ACIN1 induces apoptotic chromatin condensation [73]. This suggests that NCDN depletion may promote apoptotic pathways in CRC.

In summary, this study sheds light on the functional role of the dark protein NCDN in CRC progression through an integrative proteomic approach. We demonstrate the importance of NCDN in CRC, supported by its strong expression correlation with PODXL and ERM proteins. NCDN depletion significantly impaired cell attachment, suggesting its involvement in tumorigenic and metastatic properties mediated by the PODXL-ezrin axis. This effect appears to be orchestrated through specific intermediary factors identified in this study, including desmosomal proteins, talin-2, and SLC25A5. Moreover, our findings indicate that NCDN influences additional cellular processes beyond the PODXL-ezrin pathway, such as mitochondrial function, redox homeostasis, and aspects of the tumor microenvironment. Whether these effects are directly mediated by NCDN or arise from downstream regulatory changes remains to be determined. Nonetheless, our results position NCDN and its interactome as promising therapeutic targets, warranting further investigation into their multifaceted roles in CRC progression and metastasis.

## Supplementary information

Supplementary Figure 1

Supplementary Figure 2

Supplementary Figure 3

Supplementary Table S1

Supplementary Table S2

Supplementary Table S3

Supplementary Table S4

Uncropped images

## Data Availability

All data generated or analyzed during this study are included in this published article and its supplementary information files. The Mass Spectrometry data were deposited to the ProteomeXchange Consortium via the PRIDE partner repository with the dataset identifier PXD061002 (TMT) and PXD066531 (IP).
