# Supplementary material for: Neurochondrin drives colorectal cancer progression by modulating the PODXL–Ezrin axis and mitochondrial function
Source: Cell Death Dis. 2026 Apr 17;17(1):511. doi: 10.1038/s41419-026-08747-5 (PMC13216627; doi:10.1038/s41419-026-08747-5)
Supplement: Supplementary file 1 — Supplementary Figure 1 [file 41419_2026_8747_MOESM1_ESM.pptx]

## Slide 1
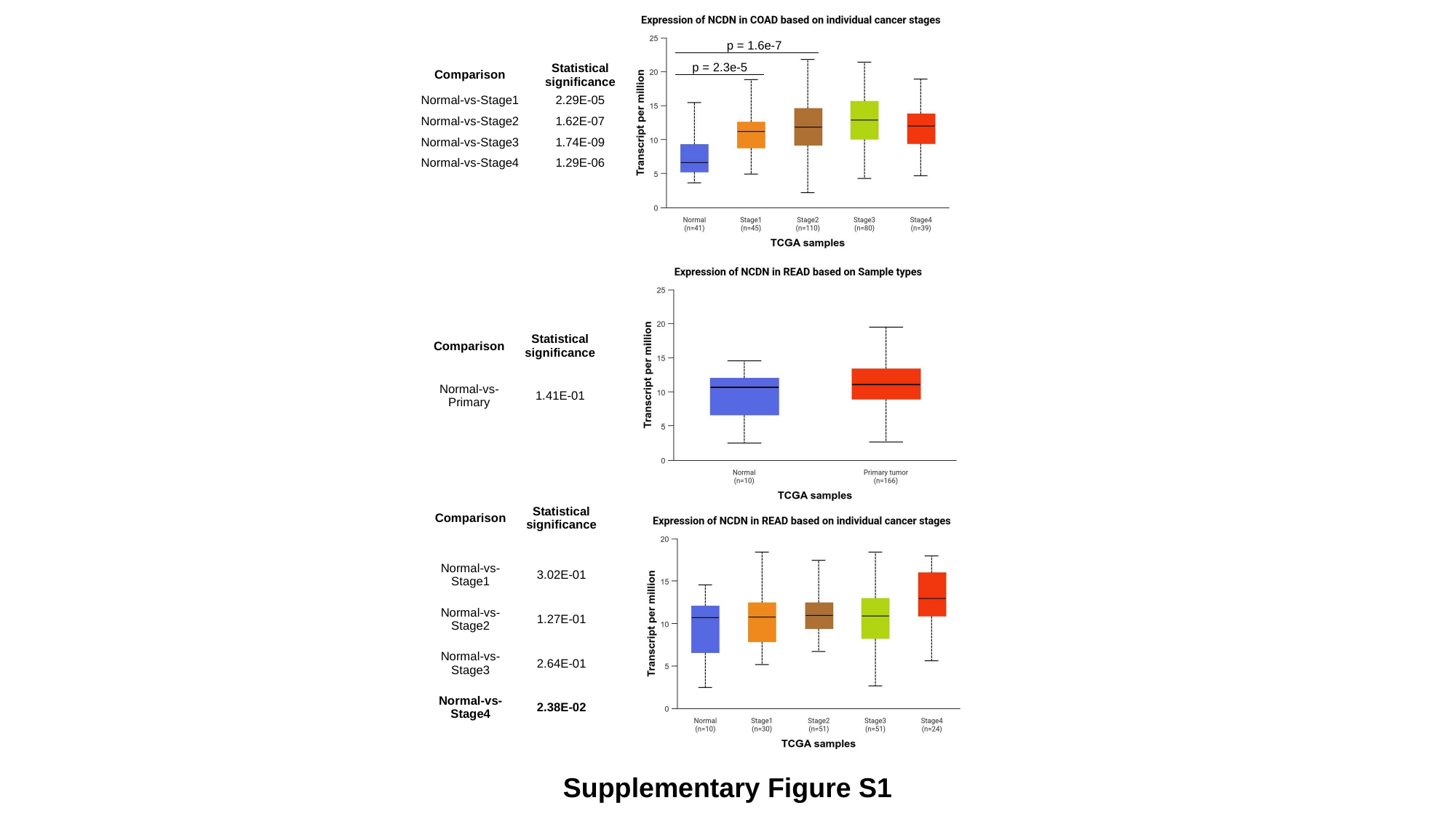

p = 1.6e-7
p = 2.3e-5
| Comparison | Statistical significance |
| --- | --- |
| Normal-vs-Stage1 | 2.29E-05 |
| Normal-vs-Stage2 | 1.62E-07 |
| Normal-vs-Stage3 | 1.74E-09 |
| Normal-vs-Stage4 | 1.29E-06 |
| Comparison | Statistical significance |
| --- | --- |
| Normal-vs-Primary | 1.41E-01 |
| Comparison | Statistical significance |
| --- | --- |
| Normal-vs-Stage1 | 3.02E-01 |
| Normal-vs-Stage2 | 1.27E-01 |
| Normal-vs-Stage3 | 2.64E-01 |
| Normal-vs-Stage4 | 2.38E-02 |
Supplementary Figure S1
