# Supplementary material for: Neurochondrin drives colorectal cancer progression by modulating the PODXL–Ezrin axis and mitochondrial function
Source: Cell Death Dis. 2026 Apr 17;17(1):511. doi: 10.1038/s41419-026-08747-5 (PMC13216627; doi:10.1038/s41419-026-08747-5)
Supplement: Supplementary file 2 — Supplementary Figure 2 [file 41419_2026_8747_MOESM2_ESM.pptx]

## Slide 1
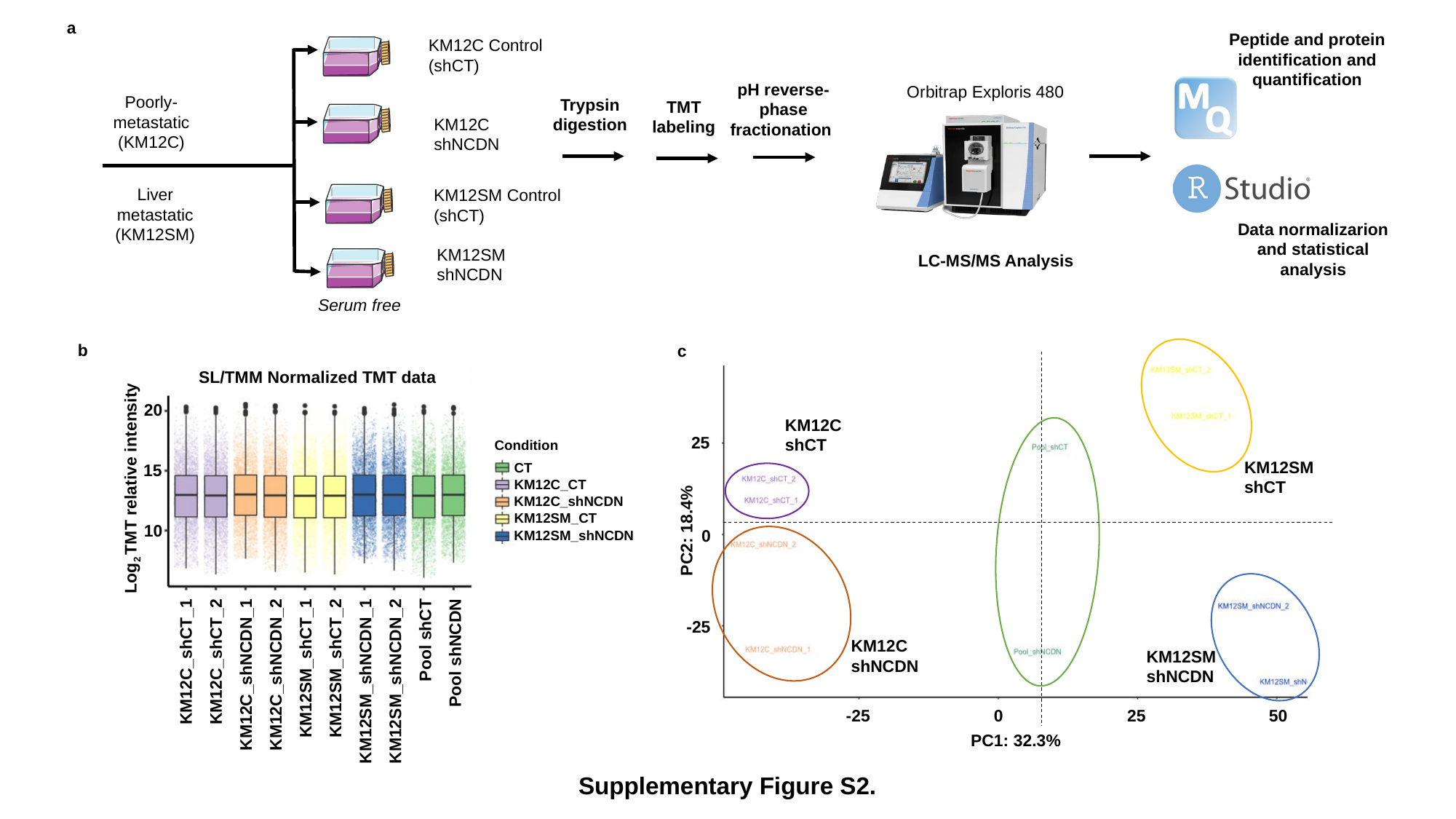

a
Peptide and protein identification and quantification
KM12C Control (shCT)
pH reverse-phase fractionation
Orbitrap Exploris 480
LC-MS/MS Analysis
Poorly-metastatic (KM12C)
Trypsin digestion
TMT labeling
KM12C shNCDN
Liver metastatic (KM12SM)
KM12SM Control (shCT)
Data normalizarion and statistical analysis
KM12SM shNCDN
Serum free
b
KM12C shCT
KM12SM shCT
KM12C shNCDN
KM12SM shNCDN
c
SL/TMM Normalized TMT data
20
25
Condition
CT
15
KM12C_CT
Log2 TMT relative intensity
KM12C_shNCDN
KM12SM_CT
PC2: 18.4%
10
0
KM12SM_shNCDN
-25
KM12C_shCT_1
KM12C_shCT_2
KM12SM_shCT_1
KM12C_shNCDN_2
KM12SM_shCT_2
KM12C_shNCDN_1
KM12SM_shNCDN_1
KM12SM_shNCDN_2
Pool shCT
Pool shNCDN
-25
0
25
50
PC1: 32.3%
Supplementary Figure S2.
