# Supplementary material for: Neurochondrin drives colorectal cancer progression by modulating the PODXL–Ezrin axis and mitochondrial function
Source: Cell Death Dis. 2026 Apr 17;17(1):511. doi: 10.1038/s41419-026-08747-5 (PMC13216627; doi:10.1038/s41419-026-08747-5)
Supplement: Supplementary file 8 — Uncropped images [file 41419_2026_8747_MOESM8_ESM.pdf]

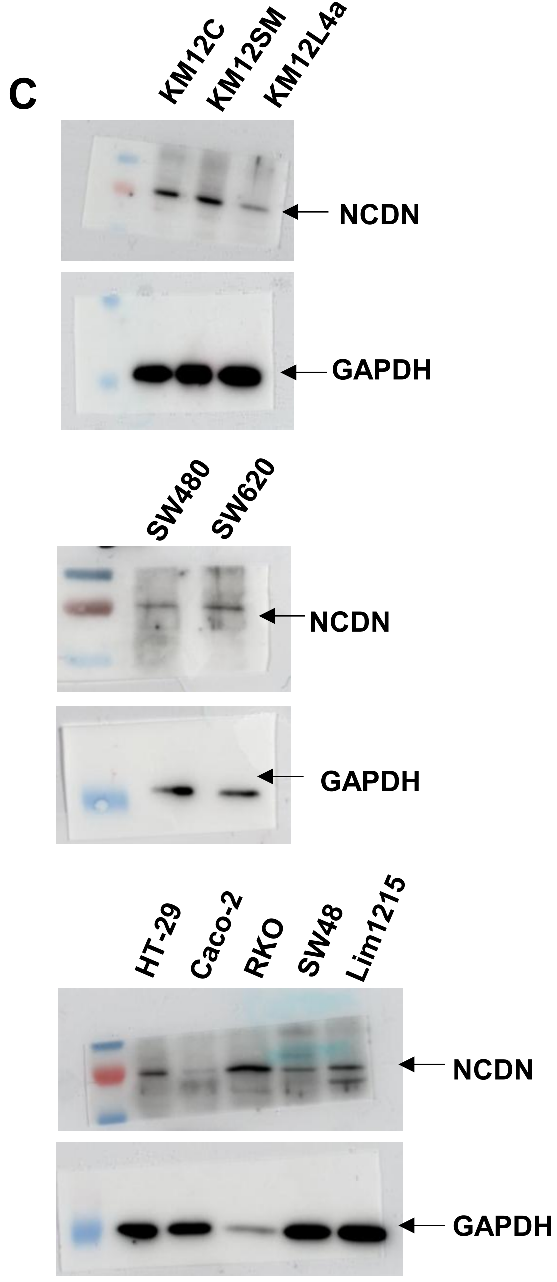

Uncropped Images
Figure 1

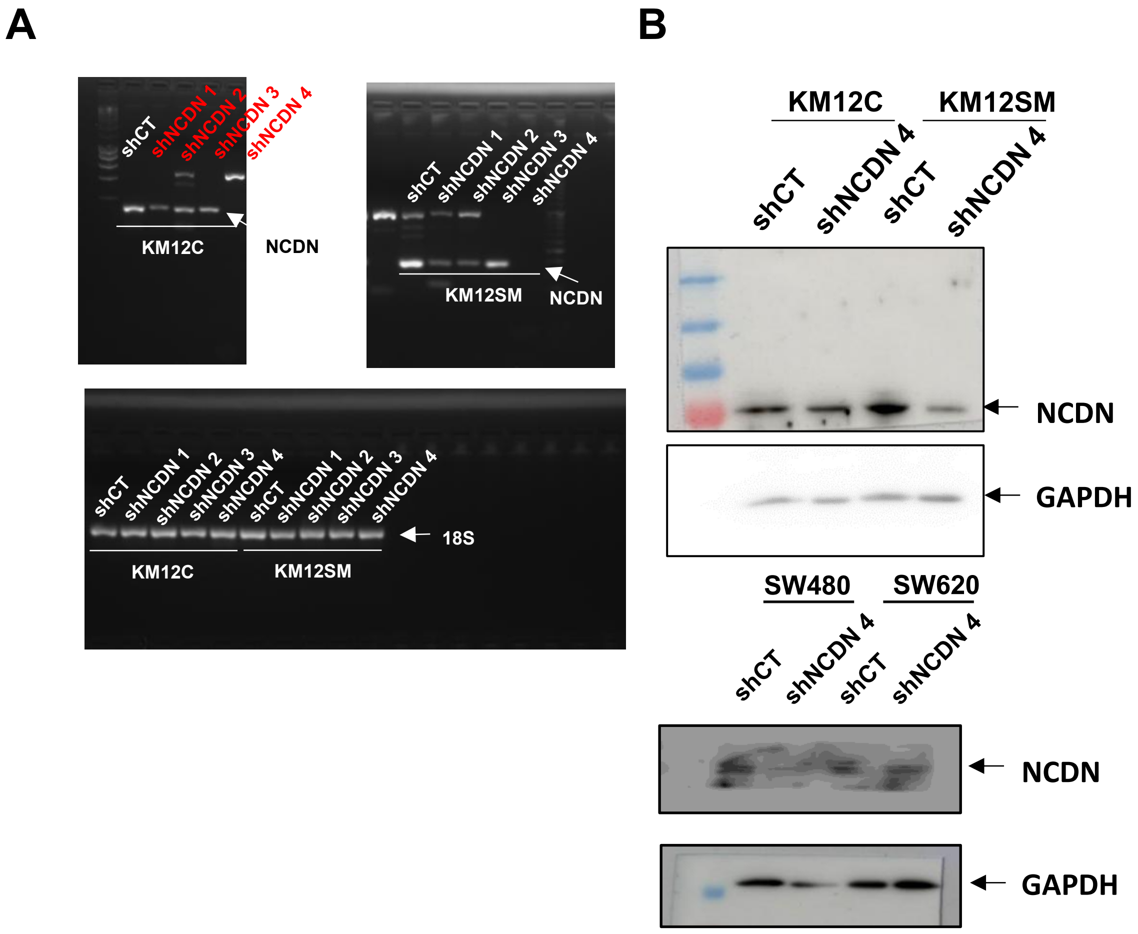

Uncropped Images Figure 2

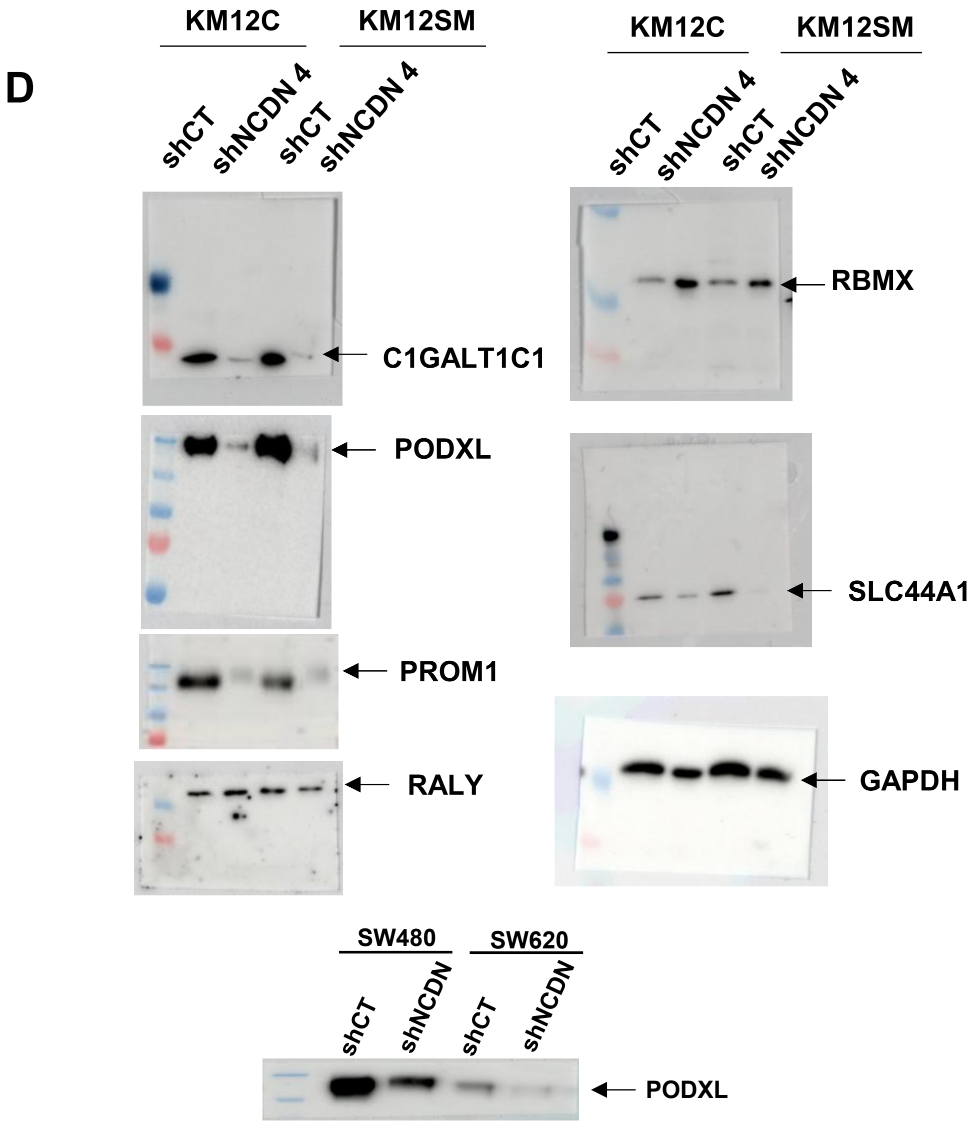

Uncropped Images Figure 4

C

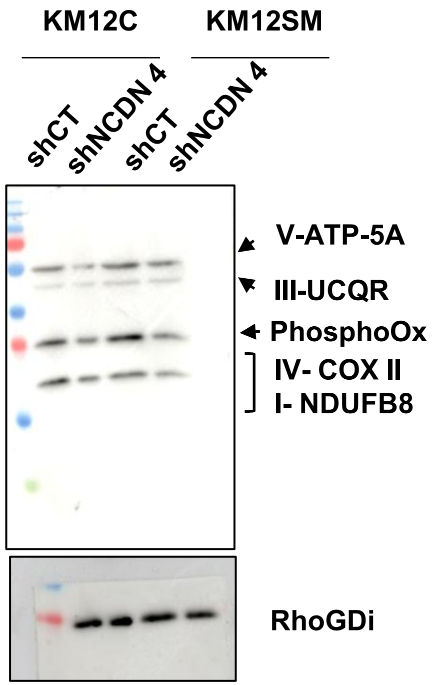

Uncropped Images
Figure 5

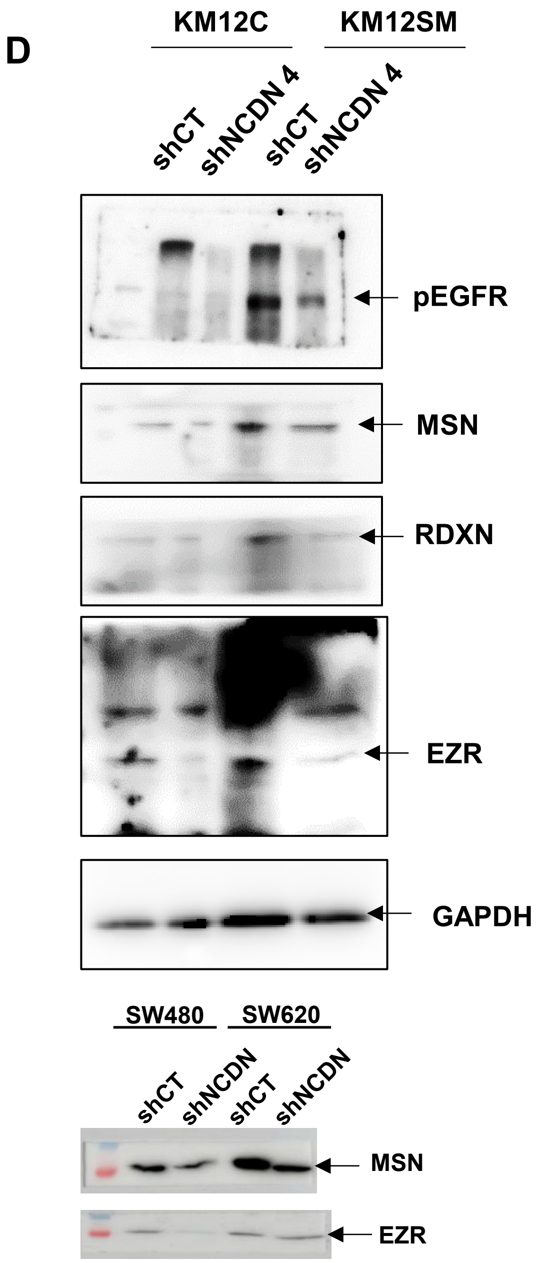

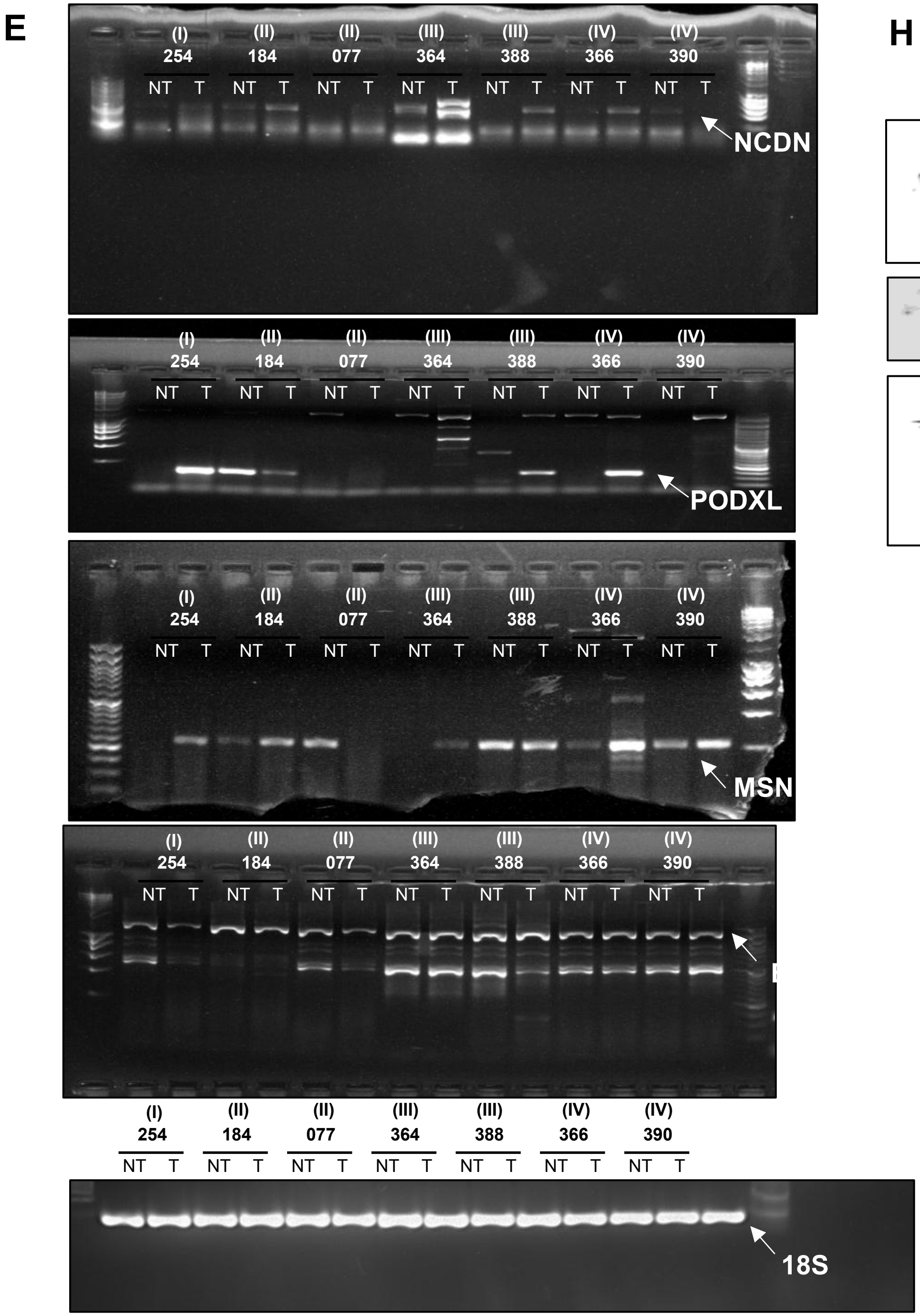

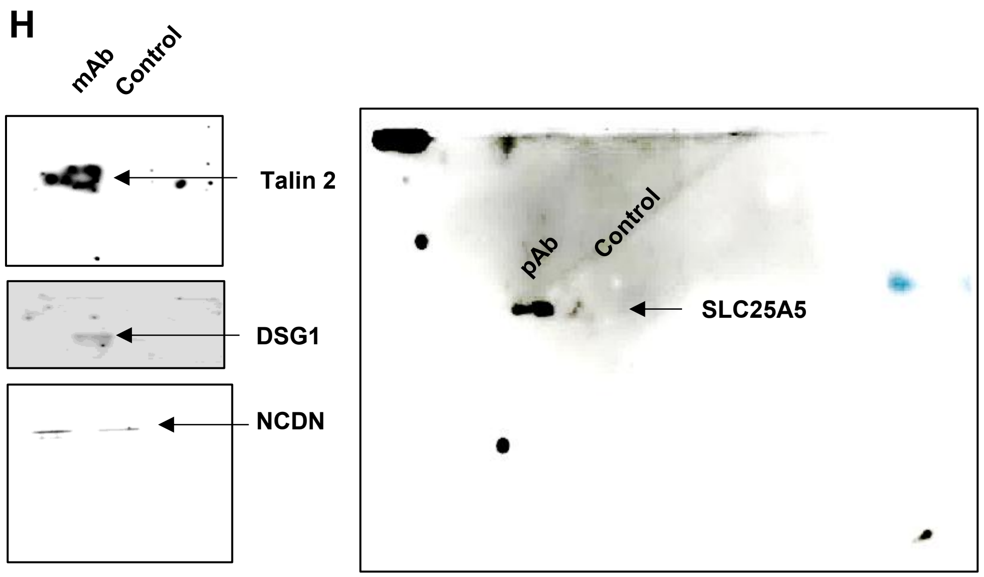

Uncropped Images
Figure 6
